# Compact Neural Architecture Designs by Tensor Representations

**DOI:** 10.3389/frai.2022.728761

**Published:** 2022-03-08

**Authors:** Jiahao Su, Jingling Li, Xiaoyu Liu, Teresa Ranadive, Christopher Coley, Tai-Ching Tuan, Furong Huang

**Affiliations:** ^1^Department of Electrical and Computer Engineering, University of Maryland, College Park, MD, United States; ^2^Department of Computer Science, University of Maryland, College Park, MD, United States; ^3^Laboratory for Physical Sciences, University of Maryland, College Park, MD, United States; ^4^Department of Aeronautics, United States Air Force Academy, Colorado Springs, CO, United States

**Keywords:** tensor decomposition, tensor networks, neural networks, deep learning, model compression

## Abstract

We propose a framework of tensorial neural networks (TNNs) extending existing linear layers on low-order tensors to multilinear operations on higher-order tensors. TNNs have three advantages over existing networks: First, TNNs naturally apply to higher-order data without flattening, which preserves their multi-dimensional structures. Second, compressing a pre-trained network into a TNN results in a model with similar expressive power but fewer parameters. Finally, TNNs interpret advanced compact designs of network architectures, such as bottleneck modules and interleaved group convolutions. To learn TNNs, we derive their backpropagation rules using a novel suite of generalized tensor algebra. With backpropagation, we can either learn TNNs from scratch or pre-trained models using knowledge distillation. Experiments on VGG, ResNet, and Wide-ResNet demonstrate that TNNs outperform the state-of-the-art low-rank methods on a wide range of backbone networks and datasets.

## 1. Introduction

Modern neural networks (Krizhevsky et al., 2012; Simonyan and Zisserman, 2014; He et al., 2016b; Zagoruyko and Komodakis, 2016; Huang et al., 2017; Szegedy et al., 2017) achieve unprecedented performance on many difficult learning problems at the cost of requiring excessive model parameters for deeper and wider architectures. The vast number of model parameters is a practical obstacle to deploying neural networks on constrained devices, such as smartphones and IoT devices. Thus a fundamental problem in deep learning is to design neural networks with compact architectures that maintain expressive power comparable to large models. Two complementary approaches are common for this purpose: one compresses pre-trained models while preserving their performance as much as possible (Cheng et al., 2017); the other aims to develop compact neural architectures such as inception modules (Szegedy et al., 2017), interleaved group convolutions (Zhang et al., 2017), and bottleneck blocks (Lin et al., 2013; He et al., 2016b). Since linear layers (i.e., fully-connected and convolutional layers) comprise almost all parameters and computation, he common goal of both approaches is to reduce the expense by the linear operations.

Motivated by the tensor decomposition of linear layers (Lebedev et al., 2014; Kim et al., 2015; Novikov et al., 2015), we propose a framework of tensorial layers that outlines the design space of low-rank factorization the framework simultaneously allows compression of pre-trained models and exploration of better network architectures. Our proposed tensorial layers extend the linear operations of matrix multiplications (in fully-connected layers) and multi-channel convolutions (in convolutional layers) to multilinear operations with multiple kernels. To characterize these layers, we introduce a novel suite of generalized tensor algebra that extends linear operations on low-order tensors to multilinear ones on higher-order tensors (cf. section 3).

We name a neural network composed of tensorial layers as a *tensorial neural network* (TNN), which by definition generalizes the traditional neural network (NN)—if we restrict the multi-linear operations in tensorial layers to matrix multiplications or multi-channel convolutions, the TNN reduces to a traditional NN. Unlike traditional NNs that may flatten the data into low-order tensors (e.g., from videos to frames), TNNs allow for data with arbitrary order. Quite the opposite, TNNs deliberately reshape the data into higher-order tensors and use higher-order weight kernels in each layer. In this higher-order space, TNNs can achieve strong expressive power with a smaller number of parameters.

**Figure 1:**
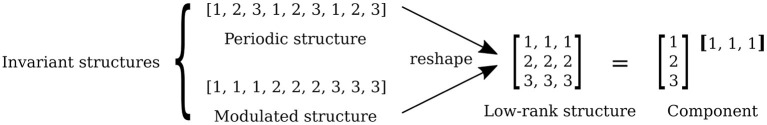
A toy example of invariant structures. The periodic and modulated structures are exposed by exploiting the low rank structure in the reshaped matrix.

To understand the benefit of higher-order space, we illustrate with a toy example in Figure 1. Consider a vector with periodic structure [1, 2, 3, 1, 2, 3, 1, 2, 3] or with modulated structure [1, 1, 1, 2, 2, 2, 3, 3, 3], representing the vector naively requires 9 parameters, which by itself cannot be further compressed by factorization. However, if we reshape the vector into a higher-order object, for instance, a matrix [1, 1, 1;2, 2, 2;3, 3, 3]. Since all columns of this matrix are the same, we can decompose the rank-1 matrix into an outer product of two vectors without losing information. Therefore, only 6 parameters are needed to represent the original length-9 vector. Intuitively, it is easier to represent higher-order tensors in a factorized form than low-order ones.

To use TNNs in practice, we need to address both *prediction* and *learning* problems in tensorial layers. (1) Prediction with a TNN is similar to a traditional NN: its input passes through all layers in a feedforward manner. In a TNN, each layer involves a generalized tensor operation between the *higher-order* input and multiple weight kernels, followed by an activation function such as ReLU. (2) To provide a practical solution to the learning problem, we derive efficient backpropagation rules (Rumelhart et al., 1986) for a broad family of tensorial layers using the newly introduced tensor algebra. We can then efficiently learn TNNs using first-order optimization methods such as stochastic gradient descent (SGD).

Although we could build and train TNNs from scratch, we can also use them to compress pre-trained NNs, as tensorial layers naturally identify both *low-rank* and *invariant* structures in the original kernels of the linear layers (Figure 1). Given a pre-trained NN $g^{q}\in G^{q}$ with *q* parameters, we may compress it to a TNN $h^{p}\in H^{p}$ with *p* parameters as depicted in **Figure 6**. This process involves two steps: **(1) data tensorization**: reshaping the input into a higher-order tensor; and **(2) knowledge distillation**: mapping a NN to a TNN, using layer-wise data reconstruction.

We demonstrate the expressive power of TNNs by conducting experiments on several benchmark image classification datasets. Our algorithm compresses ResNet-32 on the CIFAR-10 dataset by 10× with degradation of only 1.92% (achieving an accuracy of 91.28%). Experiments on LeNet-5, VGG, ResNet, and Wide-ResNet consistently verify that our tensorial neural networks outperform the state-of-the-art low-rank architectures under the same compression rate (with 5% test accuracy improvement on CIFAR-10 using sequential knowledge distillation and ImageNet when trained from scratch).

**Contributions**. In summary, we make the following contributions in this article:

1. We propose a framework of *tensorial layers*, which extends special linear operations in traditional neural networks to general multilinear operations. This results in *tensorial neural networks* (TNNs) that allow for compact architecture designs in higher-order space.
2. We introduce a system of *generalized tensor algebra*, with which we derive efficient prediction and learning in tensorial neural networks (TNNs). In particular, we are the first to derive and analyze backpropagation for generalized tensor operations.
3. We develop an effective algorithm to compress pre-trained models into tensorial neural networks (TNNs), exploiting low-rank and invariant structures in the parameter space.
4. We provide interpretations of famous network architectures with our proposed tensorial layers, explaining why these famous architectures are empirically successful. Our framework provides a principled way to design structured weight matrices/tensors (see examples in **Figures 7**, **8**).

The rest of this article is structured as follows. Section 2 gives an overview of the related works. Section 3 introduces generalized tensor operations and their representations in tensor diagrams. Based on these operations, section 4 proposes a family of tensorial layers, extending fully connected/convolutional layers in traditional neural networks. Section 6 interprets numerous compact network designs from the perspective of tensorial layers. Then section 5 provides practical algorithms to learn tensorial layers in tensorial neural networks, and section 7 demonstrate the performance of our algorithms in learning compact TNNs. Finally, section 8 concludes our contributions in this paper.

## 2. Related Work

**Tensor networks** are widely used in quantum physics (Orús, 2014), numerical analysis (Grasedyck et al., 2013), and machine learning (Cichocki et al., 2016, 2017). Cohen and Shashua (2016) and Khrulkov et al. (2018) use tensor networks to establish the expressive power of convolutional and recurrent neural networks. Recently, Hayashi et al. (2019) combine tensor networks with genetic algorithms to search for efficient layer designs. Unlike our work, the search space in Hayashi et al. (2019) only includes low-order tensors. Moreover, their method does not consider applying knowledge distillation to pre-trained models to produce more compact architectures.

**Model compression of neural networks**. Existing approaches for neural network compression can be roughly grouped into the following categories: *low-rank factorization, design of compact filters, knowledge distillation*, as well as *pruning, quantization, and encoding*.

1. *Low-rank factorization*. Various factorizations have been proposed to reduce the number of parameters in linear layers. Pioneering works propose to flattening/unfolding the parameters in convolutional layers into matrices (known as *matricization*), followed by dictionary learning or matrix decomposition (Denton et al., 2014; Jaderberg et al., 2014; Zhang et al., 2015). Subsequently, Lebedev et al. (2014) and Kim et al. (2015) show that it is possible to compress these parameter structures directly using tensor decompositions (e.g., CP or Tucker decomposition Kolda and Bader, 2009). The groundbreaking works (Novikov et al., 2015; Garipov et al., 2016) demonstrate that the low-order parameter structures can be efficiently compressed *via* tensor-train decomposition (Oseledets, 2011) by first reshaping the structures into a higher-order tensor. This idea is later extended in two directions: tensor-train decomposition is used to compress LSTM/GRU layers in recurrent neural networks (Yang et al., 2017), higher-order recurrent neural networks (Yu et al., 2017; Su et al., 2020), and 3D convolutional layers (Wang et al., 2020); other decompositions are also explored for better compression, such as tensor-ring decomposition (Zhao et al., 2016) and block-term decomposition (Ye et al., 2020).
2. *Pruning, quantization, and encoding*. The pioneering work by Han et al. (2015) proposed a three-step pipeline to compress a pre-trained model by pruning the uninformative connections, quantizing the remaining weights, and encoding the discretized parameters. These ideas are complementary to low-rank factorization—Goyal et al. (2019) demonstrated a joint use of pruning and low-rank factorization, and Lee et al. (2021) a combination of quantization and low-rank factorization.
3. *Knowledge distillation*. This process aims to transfer information from a pre-trained teacher network to a smaller student network. Ba and Caruana (2014) and Hinton et al. (2015) proposed to train the student network with the teacher network's logits (the vector before the softmax layer). Romero et al. (2014) extend this idea so that the outputs from both networks match *at each layer*, with an affine transformation.
4. *Design of compact filters*. These techniques reduce the number of parameters by imposing additional patterns on fully-connected or convolutional layers. For example, prior works restrict the matrix in a fully-connected layer to circular (Cheng et al., 2015), Toeplitz/Vandermonde/Cauchy (Sindhwani et al., 2015), or the product of special matrices (Yang et al., 2015). Historically, convolutional layers are considered to be a compact design of fully-connected layers, where spatial connections are local (thus sparse) with repeated weights. Recent works further suggest more compact convolutional layers, such as 1 × 1 *convolutional layer* (Szegedy et al., 2017; Wu et al., 2017) (where each filter is a scalar) and *depth-wise convolutional layer* (Chollet, 2017) (where connections between features are sparse).

Our approach combines two of the above approaches: (1) it uses knowledge distillation to project a pre-trained neural network onto the set of TNNs with low-rank tensor structures, and (2) it exploits these low-rank tensor structures, which naturally correspond to compact architecture designs (structured connections) and can be efficiently evaluated using generalized tensor operations. Since other compression methods such as pruning and quantization complement our approach, they may be combined with our approach to further improve performance.

## 3. Generalized Tensor Algebra

**Notation**. Bold lower case letters (e.g., **v**), bold upper case letters (e.g., **M**), and calligraphic letters (e.g., T) are used to denote vectors, matrices, and multi-dimensional arrays (tensors), respectivly. We say that the array $T\in {\mathbb{R}}^{I_{0}\times \cdots \times I_{m-1}}$ is a *m-order* tensor. Furthermore, the *k*th coordinate of the entries of T corresponds to the *k*th *mode* of T, and *I*_*k*_ is referred to as the *dimension* of T along mode-*k*. By fixing all indices of T, except that corresponding to mode-*k*, we obtain the mode-*k* *fibers* of T, so that the vector $T_{i_{0},\cdots \;,i_{k-1},:,i_{k+1},\cdots \;,i_{m-1}}\in {\mathbb{R}}^{I_{k}}$ denotes the mode-*k* fiber of T indexed by (*i*_0_, ⋯ , *i*_*k*−1_, *i*_*k*+1_, ⋯ , *i*_*m*−1_).

**Figure 2:**
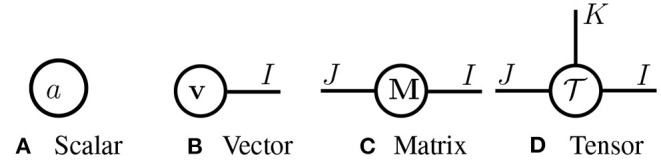
**Tensor diagrams** of **(A)** a scalar *a* ∈ ℝ, **(B)** vector **v** ∈ ℝ^*I*^, **(C)** matrix **M** ∈ ℝ^*I*×*J*^, and **(D)** tensor $T\in {\mathbb{R}}^{I\times J\times K}$.

**Tensor diagrams**. In Figure 2, we introduce *tensor diagrams*, graphical representations of multi-dimensional arrays following Grasedyck et al. (2013); Orús (2014). In tensor diagrams, each array (scalar, vector, matrix or higher-order tensor) is represented as a *node*, and its order is denoted by the number of *legs* extending from the node. Each leg corresponds to one mode of the tensor, whose dimension is denoted by an associated positive integer. Notice that tensor diagrams are *ordering-agnostic*, e.g., a matrix **M** ∈ ℝ^*I*×*J*^ and its transpose **M**^⊤^ ∈ ℝ^*J*×*I*^ have the same diagram.

**Figure 3:**
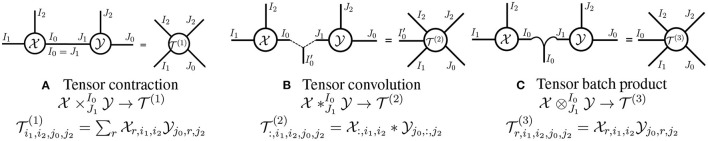
**Diagrams of primitive tensor operations**. Given $X\in {\mathbb{R}}^{I_{0}\times I_{1}\times I_{2}}$ and $Y\in {\mathbb{R}}^{J_{0}\times J_{1}\times J_{2}}$, we illustrate **(A)** $T^{\left(1\right)}=X\times_{J_{l}}^{I_{k}}Y\in {\mathbb{R}}^{I_{1}\times I_{2}\times J_{0}\times J_{2}}$, **(B)** $T^{\left(2\right)}=X{*}_{J_{l}}^{I_{k}}Y\in {\mathbb{R}}^{I_{0}^{\prime }\times I_{1}\times I_{2}\times J_{0}\times J_{2}}$, and **(C)** $T^{\left(3\right)}=X{⊗}_{J_{l}}^{I_{k}}Y\in {\mathbb{R}}^{I_{0}\times I_{1}\times I_{2}\times J_{0}\times J_{2}}$ with the above tensor diagrams.

**Primitive tensor operations**. In Table 1, we define primitives for *generalized tensor operations* on arbitrary-order tensors. In Figure 3, we illustrate these primitives using tensor diagrams. In these diagrams, a tensor operation is represented with a (hyper-)edge that links the legs of two input tensors: a solid edge denotes a tensor contraction, a dashed edge represents a tensor convolution, and a curved edge corresponds to a tensor batch product. Since tensor diagrams are ordering-agnostic, we suppress the mode indices of the tensor operations they illustrate in order to simplify notation.

**Table 1:** Primitive tensor operations.

**Operator**	**Notation**	**Definition**
mode-(*k, l*) Tensor Contraction	$T^{\left(1\right)}=X\times_{\left(l,J_{l}\right)}^{\left(k,I_{k}\right)}Y$ $\;=X\times_{J_{l}}^{I_{k}}Y$	$T_{i_{0},\cdots \;,i_{k-1},i_{k+1},\cdots \;,i_{m-1},j_{0},\cdots \;,j_{l-1},j_{l+1},\cdots \;,j_{n-1}}^{\left(1\right)}$ $=X_{i_{0},\cdots \;,i_{k-1},:,i_{k+1},\cdots \;,i_{m-1}},Y_{j_{0},\cdots \;,j_{l-1},:,j_{l+1},\cdots \;,j_{n-1}}$ Inner product of mode-*k* fiber of X and mode-*l* fiber of Y
mode-(*k, l*) Tensor Convolution	$T^{\left(2\right)}=X{*}_{\left(l,J_{l}\right)}^{\left(k,I_{k}\right)}Y$ $\;=X{*}_{J_{l}}^{I_{k}}Y$	$T_{i_{0},\cdots \;,i_{k-1},:,i_{k+1},\cdots \;,i_{m-1},j_{0},\cdots \;,j_{l-1},j_{l+1},\cdots \;,j_{n-1}}^{\left(2\right)}$ $=X_{i_{0},\cdots \;,i_{k-1},:,i_{k+1},\cdots \;,i_{m-1}}*Y_{j_{0},\cdots \;,j_{l-1},:,j_{l+1},\cdots \;,j_{n-1}}$ Convolution of mode-*k* fiber of X and mode-*l* fiber of Y
mode-(*k, l*) Tensor Batch Product	$T^{\left(3\right)}=X{⊗}_{\left(l,J_{l}\right)}^{\left(k,I_{k}\right)}Y$ $\;=X{⊗}_{J_{l}}^{I_{k}}Y$	$T_{i_{0},\cdots \;,i_{k-1},r,i_{k+1},\cdots \;,i_{m-1},j_{0},\cdots \;,j_{l-1},:,j_{l+1},\cdots \;,j_{n-1}}^{\left(3\right)}$ $=X_{i_{0},\cdots \;,i_{k-1},r,i_{k+1},\cdots \;,i_{m-1}}Y_{j_{0},\cdots \;,j_{l-1},r,j_{l+1},\cdots \;,j_{n-1}}$ Hadamard product of mode-*k* fiber of X and mode-*l* fiber of Y

*If $X\in {\mathbb{R}}^{I_{0}\times \cdots \times I_{m-\text{1}}}$ and $Y\in {\mathbb{R}}^{J_{0}\times \cdots \times J_{n-\text{1}}}$, then $T^{\left(\text{1}\right)}$, $T^{\left(\text{2}\right)}$, and $T^{\left(\text{3}\right)}$ are the mode-(k, l) tensor contraction, convolution, and batch product of X and Y, respectively. Note that the contraction and batch product are defined only if I_k_ = J_l_. Further, $T^{\left(\text{1}\right)}$ is an (m+n−2)-order tensor; $T^{\left(\text{2}\right)}$, $T^{\left(\text{3}\right)}$ are (m+n−1)-order tensors. Finally, we suppress the mode indices when expressing any of the above operations between tensors using mode ordering-agnostic tensor diagrams (cf. Figure 3)*.

**Figure 4:**
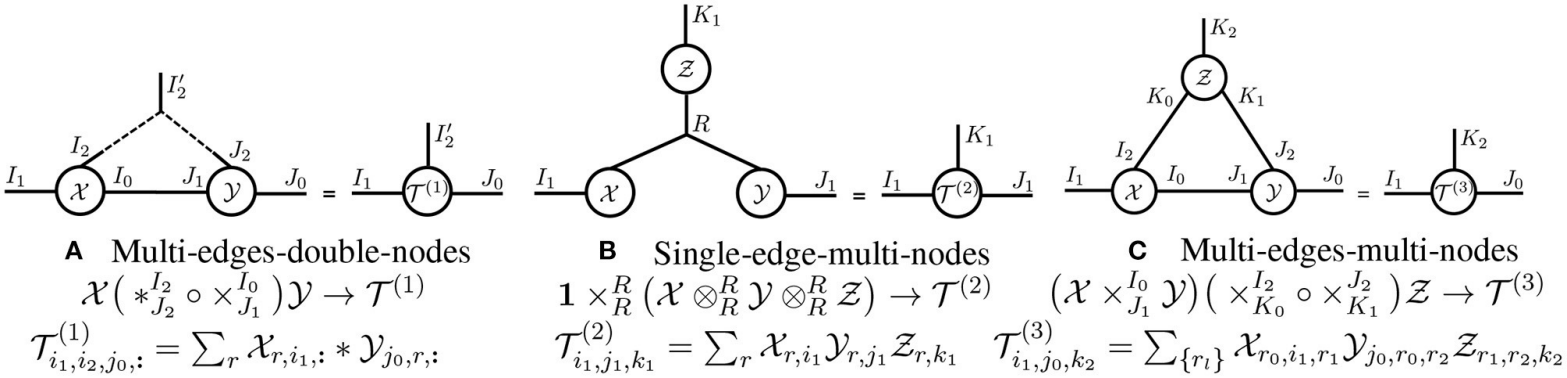
**Generalized tensor operation diagrams**. Generalized tensor operations apply one or more primitive tensor operations to two or more tensors. The above tensor diagrams illustrate three different generalized tensor operations, which represent **(A)** a 1D-convolutional layer from a neural network, **(B)** a CP-tensor decomposition, and **(C)** a tensor-ring decomposition.

**Generalized tensor operations**. Generalized tensor operations take two or more tensors as inputs and carry out one or more primitive operations on those tensors. In Figure 4, we illustrate three non-primitive generalized tensor operations. We refer to the primitive tensor operations in Figure 3 as *single-edge-double-node* operations; similarly, the three generalized tensor operations in Figure 4 are called *multi-edge-double-node, single-edge-multi-node*, and *multi-edge-multi-node* operations, respectively. Given a generalized tensor operation formed from more than one primitive operation, we may evaluate the primitives in any order to obtain the same result. However, in practice, evaluating the primitives in one order may require substantially more floating point operations (FLOPs) than in another. While it is NP-hard to obtain the best order (that requires the fewest FLOPs) (Lam et al., 1997), an exhaustive search is practical if the number of input tensors is small (Pfeifer et al., 2014).

## 4. Tensorial Neural Networks (TNNs)

**Figure 5:**
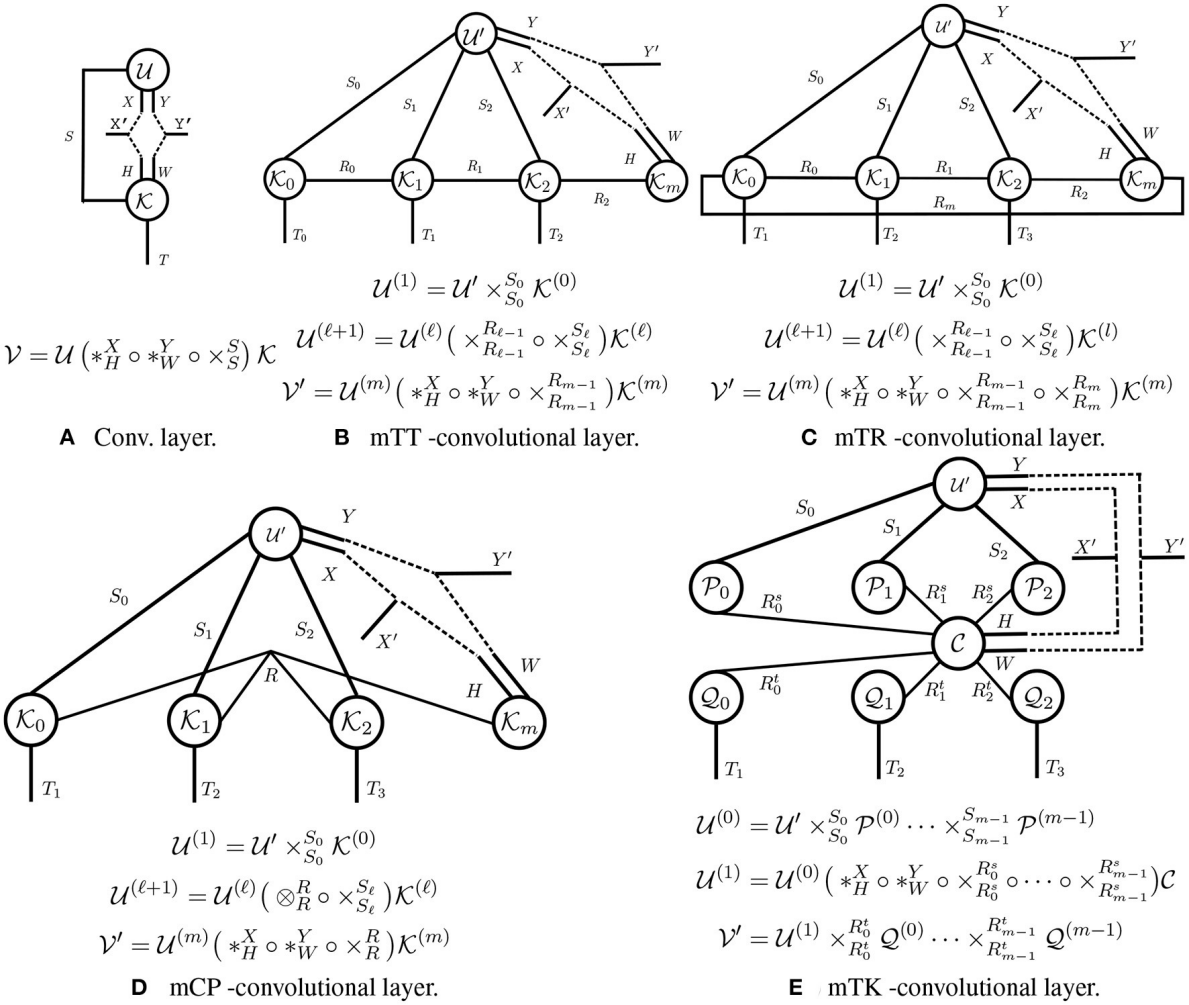
**Tensor diagrams of convolutional layers. (A)** The traditional convolutional layer is the building block for CNN; **(B–E)** The tensorial convolutional layers are building blocks for TNNs.

In this section, we introduce Tensorial Neural Networks, a type of neural network whose layers (called *tensorial layers*) are tensor networks. Tensorial layers generalize traditional fully-connected/convolutional layers, as the transformations these layers can be characterized as primitive/generalized tensor operations. For example, a fully-connected layer, which involves a matrix-vector product, is equivalent to a contraction (cf. Figure 3A), and we will see that a convolutional layer is equivalent to generalized tensor operation (cf. Figure 5A). Our primary focus is on developing tensorial layers that extend the traditional convolutional layer—since a fully-connected layer is simply a convolutional layer with filter size 1 × 1.

### 4.1. Tensorial vs. Convolutional Layers

Each layer in a convolutional neural network (CNN) is given by a compound operation applied to a 3rd-order input tensor and a 4th-order weight tensor (cf. Figure 5A). In contrast, each layer in a TNN is given by a arbitrary generalized tensor operation applied to a higher-order input tensor and *multiple* weight tensors (cf. Figure 5B). We describe both types of layers in more detail below.

**Traditional convolutional layer**. A traditional 2D-convolutional layer is parameterized by a 4th-order weight kernel $K\in {\mathbb{R}}^{H\times W\times S\times T}$, where *H* (resp. *W*) is the height (resp. width) of the filter, and *S* (resp. *T*) is the number of input (resp. output) channels. Such a layer maps a 3rd-order input tensor $U\in {\mathbb{R}}^{X\times Y\times S}$ to another 3^rd^-order output tensor $V\in {\mathbb{R}}^{X^{\prime }\times Y^{\prime }\times T}$, where *X* (resp. *Y*) is the height (resp. width) of the input feature maps, and *X*′ (resp. *Y*′) is the height (resp. width) of the output feature maps. This convolutional layer can be concisely written using our generalized tensor operations:


(1) $$\begin{array}{l} V=U\left({*}_{H}^{X}\circ {*}_{W}^{Y}\circ \times_{S}^{S}\right)K. \end{array}$$

Moreover, a convolutional layer involves a multi-edge-double-node operation, where multiple primitive tensor operations are executed along different modes. Specifically, two tensor convolutions are performed: one along the modes with dimensions *X* and *H*, and the other along the modes with dimensions *Y* and *W*; a tensor contraction along the modes with dimension *S* is also carried out.

**Tensorial layers**. Tensorial layers involve applying a generalized tensor operation to an input tensor and multiple weight kernels. We illustrate several tensorial layers in Figures 5B–E. In Figure 5B, we illustrate a tensorial layer inspired by the Tensor-Train (TT) layer (Oseledets, 2011). We will refer to this layer as a mTT-convolutional layer (the letter “m” is for “modified;” this layer is slightly different than that in Oseledets, 2011). A mTT layer takes an (*m* + 2)-order input tensor $U^{\prime }$ and returns an output tensor $V^{\prime }$ of the same order. This layer has (*m* + 1) kernels ${\left\{K_{i}\right\}}_{i=0}^{m}$ as parameters, in order to preserve the multi-dimensional structure of $U^{\prime }$. Mode-*i* of $U^{\prime }$ contracts with its corresponding kernel $K_{i}$, and interactions between modes are captured by contractions between adjacent kernels (e.g., $K_{i}$ and $K_{i+1}$). These contractions are crucial for modeling multi-dimensional transformations with high expressive power. Thus, a mTT-convolutional layer enables the multi-dimensional propagation of a higher-order input. We refer to a network with mTT-convolutional layers as a TNN-mTT. In Figures 5C–E, we develop other tensorial layers inspired by Tensor-Ring (TR), Canonical polyadic (CP), and Tucker (TK) tensor decompositions (Kolda and Bader, 2009; Zhao et al., 2016); we refer to the corresponding networks as TNN-mTR, TNN-mCP, and TNN-mTK networks, respectively.

### 4.2. Relationships Between Tensorial and Convolutional Layers

**Approximation *via* tensor decomposition**. We can use a tensorial layer to approximate to a higher-order linear layer (fully-connected or convolutional). Suppose U, K, and V in Equation (1) are reshaped into higher-order tensors $U^{\prime }$, $K^{\prime }$, and $V^{\prime }$, such that input/output channels are indexed by *m* modes (i.e., $U^{\prime }\in {\mathbb{R}}^{X\times Y\times S_{0}\times \cdots \times S_{m-1}}$, $K^{\prime }\in {\mathbb{R}}^{H\times W\times S_{0}\times \cdots \times S_{m-1}\times T_{0}\times \cdots \times T_{m-1}}$, and $V^{\prime }\in {\mathbb{R}}^{X^{\prime }\times Y^{\prime }\times T_{0}\times \cdots \times T_{m-1}}$, where $S=\prod_{i=0}^{m-1}S_{i}$ and $T=\prod_{i=0}^{m-1}T_{i}$). We then have the following relationship between $U^{\prime }$, $K^{\prime }$, and $V^{\prime }$:


(2) $$\begin{array}{l} V^{\prime }=U^{\prime }({*}_{X}^{H}\circ {*}_{Y}^{W}\circ \times_{S_{0}}^{S_{0}}\circ \cdots \circ \times_{S_{m+1}}^{S_{m+1}})K^{\prime }. \end{array}$$

For a TNN-mTT tensorial layer, the kernels ${\left\{K_{i}\right\}}_{i=0}^{m}$ correspond to factors of $K^{\prime }$, when $K^{\prime }$ can be represented with a *modified tensor-train decomposition*:


(3) $$\begin{array}{l} K^{\prime }≜K_{0}\times_{R_{0}}^{R_{0}}K_{1}\times_{R_{1}}^{R_{1}}\cdots \times_{R_{m-1}}^{R_{m-1}}K_{m}. \end{array}$$

This motivates us to compress a linear layer into a tensorial layer, and more broadly, compress a traditional NN into a compact TNN. In section 5, we will study relevant compression algorithms in detail.

**Figure 6:**
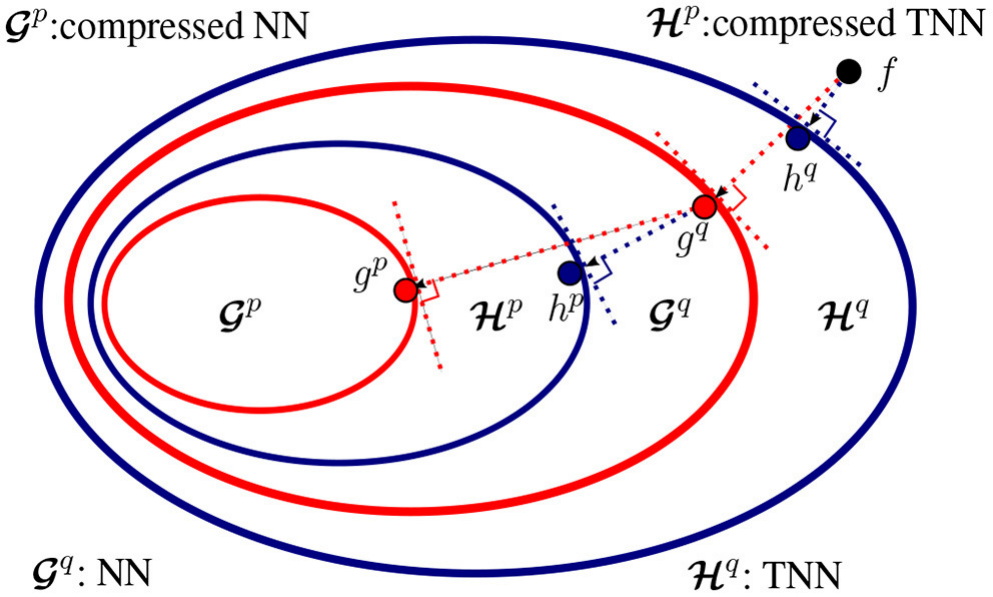
**Relationship between NNs and TNNs**. Suppose the class of NNs and TNNs have the same architecture (i.e., only the tensor operation at each layer is different), and *f* is the target concept. **(1) Learning** of a NN with *q* parameters results in *g*^*q*^ that is closest to *f* in $G^{q}$, while learning of a TNN with *q* parameters results in *h*^*q*^ that is closest to *f* in $H^{q}$. Apparently, *h*^*q*^ is closer to *f* than *g*^*q*^, **(2) Compression** of a pre-trained NN $g^{q}\in G^{q}$ to NNs with *p* parameters (*p* ≤ *q*) results in *g*^*p*^ that is closest to *g*^*q*^ in $G^{p}$, while compression of *g*^*q*^ to TNNs with *p* parameters results in *h*^*p*^ that is closest to *g*^*q*^ in $H^{p}$. Apparently, the compressed TNN *h*^*p*^ is closer to *g*^*q*^ than the compressed NN *g*^*p*^.

**Hypothesis sets of NNs and TNNs**. Suppose the class of traditional NNs and our proposed TNNs share the same architecture (i.e., only the tensor operation in each layer is different). We illustrate the relations between their hypothesis sets in Figure 6. Let $G^{q}$ and $H^{q}$ denote the classes of functions that can be represented by NNs and TNNs, both with at most *q* parameters. **(1)** *TNNs generalize NNs*. Formally, for any *q* > 0, $G^{q}\subseteq H^{q}$ holds. **(2)** *NNs can be mapped to TNNs with fewer parameters and thus TNNs can be used for compression of NNs*. Formally, there exists *p* ≤ *q* such that $H^{p}\subseteq G^{q}$.

proof: With the same backbone architecture, it suffices to prove the inclusion relations in the layer level. **(1)** is trivial. Since traditional layers are realization of tensorial layers (by setting the generalized tensor operation as a convolution or a matrix multiplication), $g^{q}\in G^{q}$ implies $g^{q}\in H^{q}$, i.e., $G^{q}\subseteq H^{q},\forall q>0$. **(2)** Let $H_{i}^{p}$ be the tensorial layer that use the *i*th type generalized tensor operation (Note that the operation types are countable), we have $H^{p}={⋃}_{i}H_{i}^{p}$. Now fix *i*, there exist *p*_*i*_ < *q* such that $H_{i}^{p_{i}}\subseteq G^{q}$ — given *p*_*i*_ small enough, any $h_{i}^{p_{i}}\in H_{i}^{p_{i}}$ can be interpreted as a factorized form of some $g^{q}\in G^{q}$ [e.g., Equation (3)]. Take $p=\underset{i}{\inf}p_{i}$, we know $H_{i}^{p}\subseteq H_{i}^{p_{i}}\subseteq G^{q},\forall i$ and therefore $H^{p}={⋃}_{i}H_{i}^{p}\subseteq G^{q}$, which completes the proof.

## 5. Algorithms for TNNs

In this section, we investigate practical algorithms for TNNs. We first develop prediction and backpropagation algorithms for TNNs, which allows us to train a TNN from scratch. We then consider algorithms that can be used to distill a compact TNN from a pre-trained model.

### 5.1. Prediction With TNNs

Prediction with TNN is similar to that of traditional neural networks: inputs are passed through layers in a feedforward manner. Each layer in a TNN involves applying a generalized tensor operation to the input and multiple weight kernels, before applying a nonlinear function such as ReLU. While it is difficult to determine the most efficient order in which to evaluate the primitives of a generalized tensor operation in general, we develop strategies to determine efficient orders for all TNN architectures introduced in this paper. For example, we can efficiently evaluate each mTT-convolutional layer as follows:


(4a) $$\begin{array}{l} U_{1}=U^{\prime }\times_{S_{0}}^{S_{0}}K_{0}, \end{array}$$

(4b) $$\begin{array}{l} U_{i+1}=U_{i}\left(\times_{R_{i-1}}^{R_{i-1}}\circ \times_{S_{i}}^{S_{i}}\right)K_{i}, \end{array}$$

(4c) $$\begin{array}{l} V^{\prime }=U_{m}\left({*}_{H}^{X}\circ {*}_{W}^{Y}\circ \times_{R_{m-1}}^{R_{m-1}}\right)K_{m}. \end{array}$$

Here, $U^{\prime }$ is the layer input, and $V^{\prime }$ is the output. The tensors ${\left\{U_{i}\right\}}_{i=1}^{m}$ are intermediate results. We provide efficient strategies for performing the forward pass in the other tensorial layers displayed in Figure 5 and Appendix B. We also summarize the complexity (the number of FLOPs and amount of parameter storage required) for each forward pass in Table 2.

**Table 2:** Complexities of traditional convolutional layer and various tensorial convolutional layers.

**Layer**	**O (params.)**	**O (forward ops.)**	**O (backward ops., input)**	**O (backward ops., params.)**
original	*k* ^2^ *N* ^2^	*k* ^2^ *N* ^2^ *D* ^2^	*k* ^2^ *N* ^2^ *D* ^2^	*N* ^2^ *D* ^4^
mCP	$\left(mN^{\frac{2}{m}}+k^{2}\right)R$	$\left(mN^{1+\frac{1}{m}}+k^{2}N\right)RD^{2}$	$\left(mN^{1+\frac{1}{m}}+k^{2}N\right)RD^{2}$	$\left(mN^{1+\frac{1}{m}}+D^{2}N\right)RD^{2}$
mTK	(2*mN* + *k*^2^*R*^2*m*−1^)*R*	(2*mN* + *k*^2^*R*^2*m*−1^)*RD*^2^	(2*mN* + *k*^2^*R*^2*m*−1^)*RD*^2^	(2*mN* + *R*^2*m*−1^*D*^2^)*RD*^2^
mTT	$\left(mN^{\frac{2}{m}}R+k^{2}\right)R$	$\left(mN^{1+\frac{1}{m}}R+k^{2}N\right)RD^{2}$	$\left(mN^{1+\frac{1}{m}}R+k^{2}N\right)RD^{2}$	$\left(mN^{1+\frac{1}{m}}R+D^{2}N\right)RD^{2}$
mTR	$\left(mN^{\frac{2}{m}}+k^{2}\right)R^{2}$	$\left(mN^{1+\frac{1}{m}}+k^{2}N\right)R^{2}D^{2}$	$\left(mN^{1+\frac{1}{m}}+k^{2}N\right)R^{2}D^{2}$	$\left(mN^{1+\frac{1}{m}}+D^{2}N\right)R^{2}D^{2}$

*Suppose X = Y = X′ = Y′ = D, S = T = N, H = W = k, and D≫k (cf. section 4)*.

*Remark: The number of FLOPs does not accurately reflect the actual running time on GPUs, as the existing CUDA library can not fully utilize the degree of parallelism in general tensor operations*.

### 5.2. Learning TNNs

To train a TNN *via* stochastic gradient descent, we derive backpropagation rules for each tensorial layer displayed in Figure 5. To derive such rules, we consider the partial derivatives of some loss function L with respect to the input ($\partial L/\partial U^{\prime }$) and kernel factors (e.g., ${\left\{\partial L/\partial K_{i}\right\}}_{i=0}^{m}$ in an mTT-convolutional layer), given $\partial L/\partial V^{\prime }$. As previously done for performing a forward pass, we develop efficient strategies for executing backpropagation with each type of tensorial layer. For an mTT-convolutional layer, an efficient strategy for performing backpropagation is


(5a) $$\begin{array}{l} \frac{\partial L}{\partial U_{m}}=\frac{\partial L}{\partial V^{\prime }}\left({{*}_{H}^{X^{\prime }}}^{⊤}\circ {{*}_{W}^{Y^{\prime }}}^{⊤}\right)K_{m}, \end{array}$$

(5b) $$\begin{array}{l} \frac{\partial L}{\partial K_{m}}=\frac{\partial L}{\partial V^{\prime }}\left({{*}_{X}^{X^{\prime }}}^{⊤}\circ {{*}_{Y}^{Y^{\prime }}}^{⊤}\times_{T_{0}}^{T_{0}}\circ \cdots \circ \times_{T_{m-1}}^{T_{m-1}}\right)U_{m}, \end{array}$$

(5c) $$\begin{array}{l} \frac{\partial L}{\partial U_{i}}=\frac{\partial L}{\partial U_{i+1}}\left(\times_{R_{i}}^{R_{i}}\circ \times_{T_{i}}^{T_{i}}\right)K_{i}, \end{array}$$

(5d) $$\begin{array}{l} \frac{\partial L}{\partial K_{i}}=\frac{\partial L}{\partial U_{i+1}}\\ \left(\times_{X}^{X}\circ \times_{Y}^{Y}\circ \times_{S_{0}}^{S_{0}}\circ \cdots \circ \times_{T_{i-1}}^{T_{i-1}}\circ \times_{S_{i+1}}^{S_{i+1}}\circ \cdots \circ \times_{S_{m-1}}^{S_{m-1}}\right)U_{i}, \end{array}$$

(5e) $$\begin{array}{l} \frac{\partial L}{\partial U^{\prime }}=\frac{\partial L}{\partial U_{1}}\left(\times_{R_{0}}^{R_{0}}\circ \times_{T_{0}}^{T_{0}}\right)K_{0}, \end{array}$$

(5f) $$\begin{array}{l} \frac{\partial L}{\partial K_{0}}=\frac{\partial L}{\partial U_{1}}\left(\times_{X}^{X}\circ \times_{Y}^{Y}\circ \times_{S_{1}}^{S_{1}}\circ \cdots \circ \times_{S_{m-1}}^{S_{m-1}}\right)U^{\prime }, \end{array}$$

where *^⊤^ denotes a transposed convolution. We derive efficient backpropagation strategies for the other tensorial layers displayed in Figure 5 and Appendix B, summarizing their complexities in Table 2.

**Learning from Scratch (Learn-Scratch)**. We can train any TNN from scratch (referred to as Learning from Scratch, or Learn-Scratch in short), given suitable algorithms for forward and backward passes. Since a TNN is formed by replacing each layer in a traditional NN with a tensorial layer, Learn-Scratch is as straightforward as training a traditional NN but is inefficient if we have a pre-trained reference NN.

### 5.3. Compression *via* Knowledge Distillation

Suppose we aim to compress a pre-trained neural network $g^{q}\in G^{q}$ to a model with *p* parameters, where *p*≪*q*. As is illustrated in Figure 6, $H^{p}$ is a broader class of networks than $G^{p}$, and hence our goal is to obtain the $h^{p}\in H^{p}$ that is, in some sense, closest to *g*^*q*^, rather than obtain the analogous $g^{p}\in G^{p}$. We expect that searching for such a *h*^*p*^ yields a network that outperforms the analogous *g*^*p*^ in terms of predictive accuracy. Intuitively, we aim to “project” a pre-trained NN $g\in G^{q}$ to a TNN $h^{⋆}\in H^{p}$. (Note that we omit the superscripts on *g* and *h* to simplify notation.) Denote the input to *g* as U and $U^{\prime }$ is a reshaped version of U (so that $U^{\prime }$ may be an input for *h*). our goal is to find *h*^⋆^ such that


(6) $$\begin{array}{l} h^{⋆}=\arg\underset{h\in H^{p}}{\min}\text{dist}\left(h\left(U^{\prime }\right),g\left(U\right)\right), \end{array}$$

where dist(·, ·) denotes any distance(-like) metric (e.g., the square of the ℓ_2_ distance) between the set of network outputs (the logits in classification problems). Solving Equation (6) is known as *knowledge distillation*; this process “distills” the knowledge from *g* and “instills” it into *h*^⋆^ (Hinton et al., 2015).

Because the class $H^{p}$ of TNNs is so vast, in practice, we minimize the objective in Equation (6), over a much smaller class of TNNs. Concretely, given the input data U and $g\in G^{q}$, we minimize the objective over the class of TNN-mTTs, TNN-mTRs, TNN-mCPs, and TNN-mTKs, where we assign each of these models a pre-specified number of layers, kernels per layer, and kernel dimensions, with a total of *p* parameters. Given a model in the class of TNNs selected, let ${\left\{K_{i}^{\left(\ell \right)}\right\}}_{i=0}^{m}$ denote the set of (*m* + 1) kernels of the ℓ^*th*^ layer of that model (replace *m* with 2*m* for TNN-mTKs). Our goal is to now search for kernels ${\left\{K_{i}^{\left(\ell \right)}\right\}}_{i,\ell }$ for all *L* layers in the TNN, such that these kernels can be used to construct the TNN *h* that is a good approximation to *g*. Specifically, we aim to solve


(7) $$\begin{array}{l} {\left\{{K_{i}^{\left(\ell \right)}}^{⋆}\right\}}_{i,\ell }=\arg\underset{{\left\{K_{i}^{\left(\ell \right)}\right\}}_{i,\ell }}{\min}\text{dist}\left(g\left(U\right),h\left(U^{\prime };{\left\{K_{i}^{\left(\ell \right)}\right\}}_{i,\ell }\right)\right). \end{array}$$

Here, dist denotes a distance metric, which we assume as the squared ℓ_2_ distance in this work. In what follows, we discuss three different approaches for solving Equation (7).

**Layer-wise Decomposition (Layer-Decomp)**. Given the relationship between TNNs and NNs (cf. section 4.2), we might solve Equation (7) with the following two steps: **(1)** For each layer (e.g., layer ℓ), we reshape the original kernel $K^{\left(\ell \right)}$ of *g* into a higher-order tensor ${K^{\prime }}^{\left(\ell \right)}$, and **(2)** we solve ${\left\{K_{i}^{\left(\ell \right)}\right\}}_{i}$ such that applying corresponding tensor operation to those kernels produces the best approximate of ${K^{\prime }}^{\left(\ell \right)}$ (we assume that $K^{\left(\ell \right)}$ is reshaped in a way such that the dimensions of ${K^{\prime }}^{\left(\ell \right)}$ match the ones of the approximate). For a mTT-convolutional layer, the second step amounts to solving the following optimization problem.


(8) $$\begin{array}{l} {\left\{{K_{i}^{\left(\ell \right)}}^{⋆}\right\}}_{i}=\arg\underset{{\left\{K_{i}^{\left(\ell \right)}\right\}}_{i}}{\min}∥{K^{\prime }}^{\left(\ell \right)}-mTT\left({\left\{K_{i}^{\left(\ell \right)}\right\}}_{i}\right){∥}^{2},\;\forall \ell , \end{array}$$

where $mTT\left({\left\{K_{i}^{\left(\ell \right)}\right\}}_{i}\right)$ denotes the result of the generalized tensor operation in Figure 5B on ${\left\{K_{i}^{\left(\ell \right)}\right\}}_{i}$ (we can formulate similar problems for other tensorial layers). Typically, one solves Equation (8) *via* an alternating least squares method (Comon et al., 2009), as Equation (8) reduces to solving a least squares problem if we fix all but one kernel in each layer. However, such a method typically does not yield accurate solutions to Equation (7). Thus, we usually only use it to initialize parameters for more advanced approaches.

**End-to-end Knowledge Distillation (E2E-KD)**. A second approach to solving Equation (7) is end-to-end knowledge distillation (E2E-KD in short), which uses stochastic gradient descent (SGD) to optimize the objective in Equation (7) over all the kernels at once. However, this approach has two main drawbacks: (1) backpropagation is expensive, as it requires end-to-end gradient flow in a TNN; and (2) SGD becomes unstable when we solve for all parameters in all layers simultaneously. To avoid these challenges, we consider the following third approach.

**Sequential Knowledge Distillation (Seq-KD)**. This third approach involves splitting Equation (7) into *L* sub-problems that we solve sequentially. Given the data input U and the network *g*, let $V^{\left(\ell \right)}$ denote its ℓ^th^ layer's output. Additionally, given the reshaped input data $U^{\prime }$, a TNN, and its kernels ${\left\{K_{i}^{\left(k\right)}\right\}}_{i,k}$, let $h_{\ell }\left({\left\{{U^{\prime }}^{\left(\ell -1\right)};K_{i}^{\left(\ell \right)}\right\}}_{i,\ell }\right)$ denote its ℓ^th^ layer's output. For the ℓ^th^ sub-problem, we assume the kernels ${\left\{K_{i}^{\left(k\right)}\right\}}_{i,k}$ are fixed for *k* < ℓ and we obtain the kernels ${\left\{K_{i}^{\left(\ell \right)}\right\}}_{i}$ by solving


(9) $$\begin{array}{l} {\left\{{K_{i}^{\left(\ell \right)}}^{⋆}\right\}}_{i}=\arg\underset{{\left\{K_{i}^{\left(\ell \right)}\right\}}_{i}}{\min}∥V^{\left(\ell \right)}-h_{\ell }\left(U^{\prime };{\left\{K_{i}^{\left(k\right)}\right\}}_{i,\ell }\right){∥}^{2}. \end{array}$$

Note that the input to the ℓ^th^ layer of either the original or compact tensor is given by the output from layer (ℓ−1), i.e., $U^{\left(\ell \right)}=V^{\left(\ell -1\right)}$ and ${U\prime }^{\left(\ell \right)}={V\prime }^{\left(\ell -1\right)}$. We solve Equation (9) using SGD, after deriving backpropagation rules for the generalized tensor operation used in the ℓ^th^ layer of the compressed TNN. Since the ℓ^th^ sub-problem depends on the result from all previous sub-problems, we must solve these problems sequentially, beginning with the layer indexed by one, and ending with the layer indexed by *L*.

## 6. Interpretation of Existing Compact Architectures

Recent advances in compact architecture designs such as *Inception* (Szegedy et al., 2017), *Exception* (Chollet, 2017), *interleaved group convolutions* (Zhang et al., 2017), and *bottleneck structures* (Lin et al., 2013; He et al., 2016b) propose to group multiple primitive operations into modules. We will show that we can express all such modules using the framework of tensorial layers (with minor modifications).

**Figure 7:**
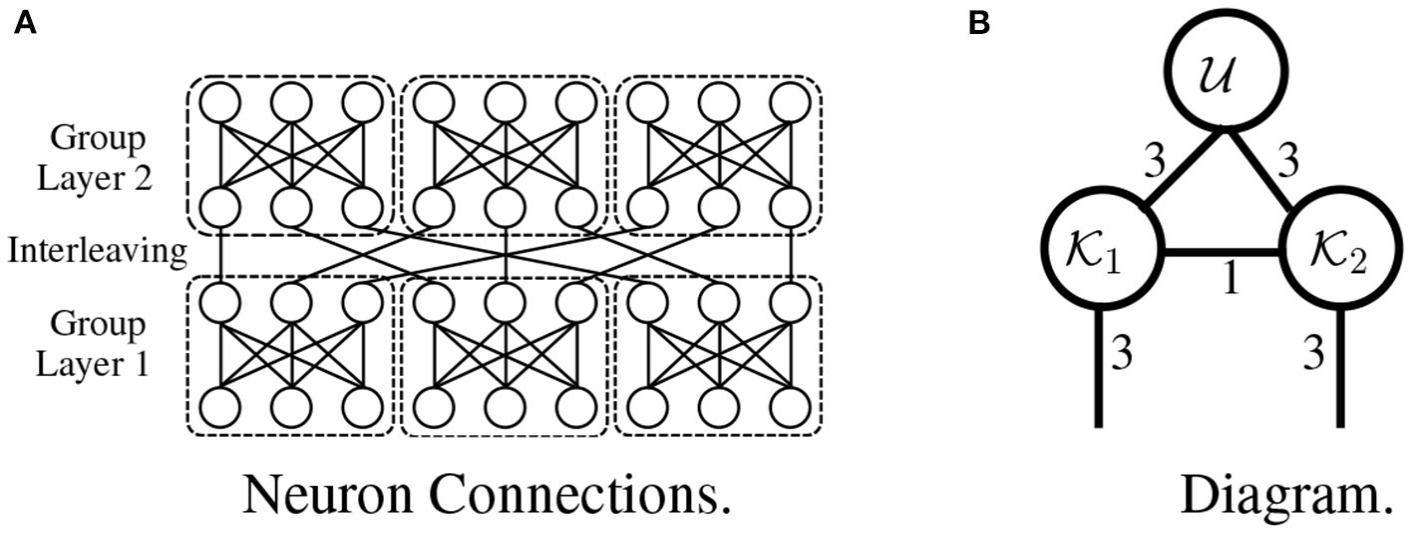
An interleaved group module without nonlinearity **(A)** is expressed as a tensorial layer **(B)**.

**Interleaved group modules**. The critical idea in interleaved group modules involves dividing and branching the input into several blocks and constraining each block's connections, which avoids computations across blocks. The architectures of tensorial layers utilize a similar strategy: for example, the tensorial layer in Figure 7B has the same architecture as the network in Figure 7A, where each length-nine input is divided into three blocks, and connections exist *only* within each block. This idea of grouping operations plays a vital role in the development of Inception (Szegedy et al., 2017) and Xception (Chollet, 2017).

**Figure 8:**
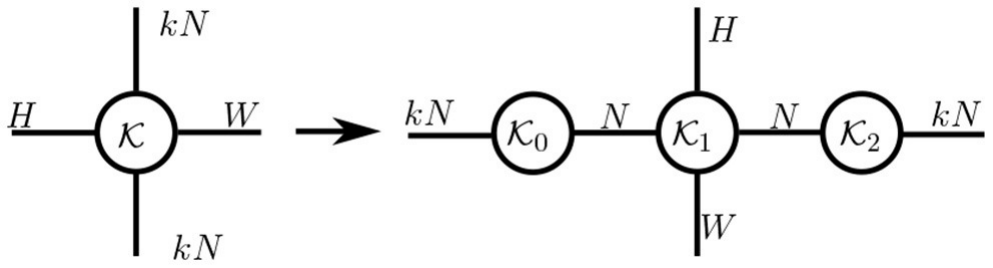
A bottleneck module without nonlinearity is expressed as a Tucker decomposition of the original layer.

**Bottleneck modules**. A bottleneck structure forces a model to adopt a compact representation by constructing a narrow bottleneck (with fewer hidden units) in the middle of each module. Such modules correspond to the low-rank structures used in tensorial layers, as illustrated by the following example with matrices: consider a weight matrix ***W*** ∈ ℝ^*S*×*S*^, its low-rank decomposition ***W*** = ***PQ*** (with ***P*** ∈ ℝ^*S*×*R*^ and ***Q*** ∈ ℝ^*R*×*S*^). This model requires an input vector ***u*** ∈ ℝ^*S*^ to first be multiplied by ***P*** and then by ***Q*** during a forward pass. Therefore, the input ***u*** is mapped into a low-dimensional space ℝ^*R*^ after being multiplied by ***P***, resulting in a bottleneck in this two-steps module. In practice, the bottleneck module in Lin et al. (2013) and He et al. (2016b) can be represented by tensor diagrams (cf. Figure 8), whose input with *kN* channels is first mapped to a structure with *N* channels by kernel $K_{0}$.

**Discussion of compact architecture designs**. The two examples above illustrate one way of designing compact tensorial layers. This design process starts with a traditional layer (fully-connected or convolutional), followed by (optional) reshaping and some tensor decomposition of the (reshaped) kernel. Consequently, the original layer is transformed into a tensorial layer with a compact structure. We can also design novel architectures from scratch (cf. section 3), by, for example, using tensor networks as building blocks for other architectures. One recent attempt that applies this methodology is Yu et al. (2017), where *tensor-train networks* are used to introduce multilinear operations to an RNN.

## 7. Experiments

This section is divided into two parts. In section 7.1, we use pre-trained models to evaluate the effectiveness of our compression algorithms (cf. section 5.3). In section 7.2, we demonstrate that our tensorial neural networks can be trained from scratch (i.e., without reference models) on a wide range of datasets and backbone models. In both scenarios, we show that our TNNs maintain high accuracy, even when they utilize significantly fewer parameters than traditional neural networks.

**Considerations for TNN experiments**. There are three items we consider when designing the experiments with TNNs that follow: (1) Kernel Reshaping. We refer to an architecture whose kernels are reshaped into higher-order tensors (before performing a low-rank kernel factorization) as a TNN; we refer to an architecture whose kernels are factorized without reshaping as an NN. Although the latter is also a TNN, we still call it a NN, as the resulting architecture (after low-rank factorization) consists only of low-order operations (i.e., matrix multiplications and multi-channel convolutions), as in traditional neural networks. In what follows, we will compare the performance of TNNs to that of NNs. (2) Types of tensor networks. Existing NN baselines are networks that do not involve any kernel reshaping and use classical kernel decompositions, e.g., SVD (Denton et al., 2014; Jaderberg et al., 2014), CP (Denton et al., 2014; Lebedev et al., 2014), and TK (Kim et al., 2015). Therefore, we refer to these architectures as NN-SVD, NN-CP, and NN-TK architectures, where the suffix denotes the type of kernel decomposition. As discussed in section 4, we may use kernel reshaping and other types of decompositions to obtain TNNs, which achieve better expressive power than NNs (cf. Figure 6). Consequently, we refer to these architectures that involve reshaping kernels as TNN-mCPs, TNN-mTTs, TNN-mTRs, etc. (3) Training or compression strategy. We train the above models either *via* knowledge distillation or from scratch. To distinguish these two strategies, we use the term *compression* for knowledge distillation (i.e., there exists a pre-trained reference network to *compress*). We use the term *TNN-based compression* (TNN-C) to describe the process of training the TNN-mCPs, TNN-mTTs, TNN-mTRs, etc. *via* knowledge distillation, and the term *low-rank compression* (NN-C) to describe the analogous process for training the NN-SVDs, NN-CPs, NN-TKs, NN-TTs, etc.

### 7.1. Knowledge Distillation

In this part, we evaluate different algorithms of knowledge distillation in section 5.3, namely *layer-wise decomposition* (Layer-Decomp), *end-to-end knowledge distillation* (E2E-KD), and *sequential knowledge distillation* (Seq-KD). We conduct extensive experiments on compressing convolutional layers in ResNet-32 for CIFAR10, and we aim to figure out the best strategy for combining these algorithms.

**Figure 9:**
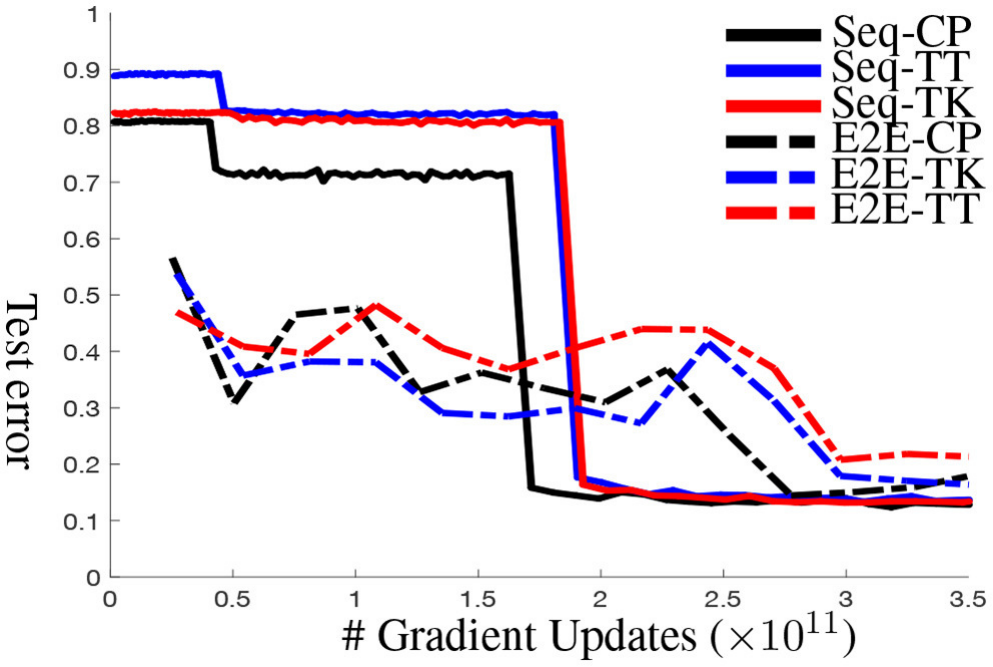
Test Error curves for **sequential knowledge distillation (Seq-KD)** vs. **end-to-end knowledge distillation (E2E-KD)** on ResNet-32 for CIFAR-10. Both approaches use **layer-wise decomposition (Layer-Decomp)** for initialization.

**Experimental setup**. We find that Layer-Decomp is merely better than random guesses in our experiments (see the test errors in Figure 9 at the beginning), Therefore, we can only use Layer-Decomp as initialization for E2E-KD and Seq-KD. With both algorithms, all layers are compressed uniformly at the same compression rate *except for the first and last layers*. Therefore, the compression rate is both layer-wise and (approximately) global. (We investigate the non-uniform allocation of all parameters across layers, but empirical results show that uniform assignment performs the best.) For all experiments, we use Adam optimizer with initial learning rate of 10^−3^, which decays by 10 every 50 epochs.

**Our algorithm achieves 5% higher accuracies than the baselines on CIFAR-10 using ResNet-32**. The results from Table 3 demonstrate that our TNNs maintain high accuracies even after the pre-trained networks are highly compressed. Given a pre-trained ResNet-32 and compression rate 10%, the NN-CP with E2E-KD reduces the original accuracy from 93.2 to 86.93%; while the TNN-mCP with Seq-KD maintains the accuracy as 91.28% with the same compression rate—a performance loss of 2% with only 10% of the number of parameters. Furthermore, TNN-C achieves further aggressive compression—a performance loss of 6% with only 2% of the number of parameters. We observe similar trends (higher compression and higher accuracy) are observed for TNN-mTT. The structure of the mTK decomposition makes TNN-mTK less effective with very high compression, since the decomposition poses a very narrow bottleneck, which may lose necessary information. Increasing the network size to 20% of the original provides reasonable performance on CIFAR-10 for TNN-mTK as well.

**Table 3:** Test accuracy of ResNet-32 on CIFAR-10 — comparison between end-to-end knowledge distillation (E2E-KD) using low-rank compression (NN-C) against sequential knowledge distillation (Seq-KD) with our TNN-based compression (TNN-C).

**Architect**.	**Compression rate**				**Architect**.	**Compression rate**			
	**5%**	**10%**	**20%**	**40%**		**2%**	**5%**	**10%**	**20%**
NN-SVD (Denton et al., 2014; Jaderberg et al., 2014)	83.09	87.27	89.58	90.85	TNN-TR (Wang et al., 2018)^†^	-	80.80^†^	-	90.60
NN-CP (Denton et al., 2014; Lebedev et al., 2014)	84.02	86.93	88.75	88.75	TNN-mCP	85.7	89.86	**91.28**	-
NN-TK (Kim et al., 2015)	83.57	86.00	88.03	89.35	TNN-TK	61.06	71.34	81.59	87.11
NN-TT (Garipov et al., 2016)^*^	77.44	82.92	84.13	86.64	TNN-mTT	78.95	84.26	87.89	-

*Test accuracy of ResNet-32 on CIFAR10*.

† *Cited from Wang et al. (2018), the accuracy of 80.8% is achieved by 6.67% compression rate*.

* *The architecture is proposed as a baseline in Garipov et al. (2016)*.

*The original ResNet-32 achieves 93.2% test accuracy with 0.46M parameters (He et al., 2016a)*.

*The bold number indicates the best performance in the table*.

**TNN-based compression, sequential knowledge distillation, or both?** Table 3 shows that *TNN-C with Seq-KD* outperforms *NN-C with traditional E2E-KD*. Now, we address the following question: is one factor (Seq-KD or TNN-C) primarily responsible for increased performance, or is the benefit due to synergy between the two?

(1) We present the accuracies of different compression methods in Table 4. Other than at very high compression rate (5% column in Table 4), Seq-KD consistently outperforms E2E-KD. In addition, Seq-KD converges faster and stabler compared to E2E-KD. Figure 9 plots the test error over the number of gradient updates for various compression methods.

**Table 4:** Test accuracy of ResNet-32 on CIFAR-10 — comparison between sequential knowledge distillation (Seq-KD) against end-to-end knowledge distillation (E2E-KD) using NN-C.

**Architect**.	**Compression rate**							
	**5%**		**10%**		**20%**		**40%**	
	**Seq**	**E2E**	**Seq**	**E2E**	**Seq**	**E2E**	**Seq**	**E2E**
NN-SVD (Denton et al., 2014; Jaderberg et al., 2014)	74.04	**83.09**	85.28	**87.27**	**89.74**	89.58	**91.83**	90.85
NN-CP (Denton et al., 2014; Lebedev et al., 2014)	83.19	**84.02**	**88.50**	86.93	**90.72**	88.75	**89.75**	88.75
NN-TK (Kim et al., 2015)	80.11	**83.57**	**86.75**	86.00	**89.55**	88.03	**91.30**	89.35
NN-TT (Garipov et al., 2016)	**80.77**	77.44	**87.08**	82.92	**89.14**	84.13	**91.21**	86.64

*The original ResNet-32 achieves 93.2% accuracy with 0.46M parameters (He et al., 2016a)*.

*The bold numbers indicate the better option between sequential knowledge distillation (Seq-KD) and end-to end knowledge (E2E-KD) for each setting*.

(2) We present the effect of different architectures on accuracy in Tables 5, **8**, **9**. **(2.1)** First, we compare TNNs with NNs *via* Seq-KD. Interestingly, as demonstrated in Table 5, if TNN-based compression is used, the test accuracy is restored for even very low compression rates^1^. **(2.2)** Second, we compare TNNs with NNs *via* Learn-Scratch. As demonstrated in **Tables 8**, **9**, TNNs outperform NNs trained using Learn-Scratch under the same number of parameters.

**Table 5:** Test accuracy of ResNet-32 on CIFAR-10 — comparison between sequential knowledge distillation (Seq-KD) for both baseline low-rank compression (NN-C) and our TNN-based compression (TNN-C).

**Architect**.	**Compression rate**		**Architect**.	**Compression rate**	
	**5%**	**10%**		**5%**	**10%**
NN-CP (Denton et al., 2014; Lebedev et al., 2014)	83.19	88.50	TNN-mCP	**89.86**	**91.28**
NN-TK (Kim et al., 2015)	**80.11**	**86.73**	TNN-mTK	71.34	81.59
NN-TT (Garipov et al., 2016)	80.77	87.08	TNN-mTT	**84.26**	**87.89**

*The original ResNet-32 achieves 93.2% accuracy with 0.46M parameters (He et al., 2016a)*.

*The bold numbers indicate the better option between traditional low-rank compression and our TNN-based compression*.

**Convergence rate**. Compared to end-to-end knowledge distillation (E2E-KD), an ancillary benefit of sequential knowledge distillation (Seq-KD) is that it is *much* faster and leads to more stable convergence. Figure 9 plots compression error over number of gradient updates for various methods (This experiment is for NN-C with 10% compression rate). There are three salient points: first, Seq-KD has very high error in the beginning while the “early” blocks are tuned (and the rest of the network is left unchanged to the values after tensor decomposition). However, as the final block is tuned (around 2 × 10^11^ gradient updates) in the figure, the errors drop to nearly a minimum immediately. In comparison, E2E-KD requires 50–100% more gradient updates to achieve stable performance. Finally, the result also shows that for each block, Seq-KD achieves convergence very quickly (and nearly monotonically), which results in the stair-step pattern since extra tuning of a block does not improve (or appreciably reduce) performance.

**Application on fully-connected layers**. We further demonstrate that our TNN-based compression can apply flexibly to fully-connected layers, in addition to convolutional layers. Notice that if we set the filter height/width (i.e., *H, W*) in any decomposition to one, the decomposition can be used to compress a fully-connected layer. Table 6 shows the results of applying TNN-based compression to various tensor decompositions on LeNet-5 (LeCun et al., 1998). The convolutional layers of the LeNet-5 network are *not* compressed nor trained in these experiments, and we use E2E-KD for knowledge distillation since there are only a few fully-connected layers at the top of the network. Table 6 shows **the fully-connected layers can be compressed to 0.2% losing only about 2% accuracy**. Furthermore, compressing the fully-connected layers to 1% of their original size reduces accuracy by less than 1%, demonstrating the extreme efficacy of TNN-based compression when applied to fully-connected neural network layers.

**Table 6:** Test accuracy of LeNet-5 on MNIST.

	**Compress. rate**		
**Architect**.	**0.2%**	**0.5%**	**1%**
TNN-mCP	97.21	97.92	98.65
TNN-mTK	97.71	98.56	98.52
TNN-mTT	97.69	98.43	98.63

*We compress all fully-connected layers using **TNN-based compression (TNN-C)** with **end-to-end knowledge distillation (E2E-KD)**. The original LeNet-5 achieves 99.31% test accuracy with 60K parameters (LeCun et al., 1998)*.

### 7.2. Learning From Scratch

While it is beneficial to have a pre-trained model as reference (see Table 7 for a comparison), there are scenarios that knowledge distillation is not applicable: (1) The pre-trained model is simply not available; (2) The model is too deep that a sequential knowledge distillation is too expensive; (3) We aim to learn TNNs with even higher expressive power than NNs. In this part, we verify that our TNNs are easily trained from scratch for a wide range of backbone models and datasets.

**Table 7:** Test accuracy of ResNet-32 on CIFAR-10—comparison between sequential knowledge distillation (Seq-KD) against learning from scratch (Learn-Scratch) using our TNNs.

**Architect**.	**Seq-KD**			**Learn-Scratch**		
	**2%**	**5%**	**10%**	**2%**	**5%**	**10%**
TNN-mCP	85.70	89.86	91.28	81.41	82.12	82.93
TNN-mTK	61.60	71.34	81.59	60.65	61.46	65.75
TNN-mTT	78.95	84.26	87.89	79.95	81.82	83.08

*The original ResNet-32 achieves 93.2% accuracy with 0.46M parameters (He et al., 2016a)*.

**Wide-ResNet for CIFAR-100**. In order to demonstrate that TNNs are compatible with other backbones (in addition to ResNet), we evaluate our TNNs with Wide-ResNet backbone (Zagoruyko and Komodakis, 2016) on the CIFAR-100 dataset. As shown in Table 8, our TNNs (in particular TNN-mTT), when trained from scratch, already outperform other state-of-the-art low-rank factorization-based methods.

**Table 8:** Test accuracy of Wide-ResNet-28-10 on CIFAR-100.

**Compress. rate**	**0.5%**	**1%**	**2%**	**5%**
NN-TT (Garipov et al., 2016)	37.02%	54.65%	52.69%	51.42%
NN-CP (Denton et al., 2014; Lebedev et al., 2014)	**40.74%**	**58.04%**	56.9%	64.83%
**Compression rate**	0.33%	**0.5%**	0.66%	**1%**
TNN-mTT	61.67%	**65.36%**	66.82%	**68.83%**

*We compare **baseline NNs** against our TNNs by **training all models from scratch** (i.e., without reference models). The original model achieves 81.25% accuracy with 36.5M parameters (Zagoruyko and Komodakis, 2016)*.

*The bold numbers indicates the performance under the same compression rate*.

**ResNet for ImageNet-2012**. To show that our TNNs scale to large datasets, we evaluate their performance on the ImageNet-2012 dataset with a ResNet-50 backbone. The results in Table 9 show that our TNNs significantly outperform the low-rank factorization-based methods at each compression rate. Furthermore, our TNNs maintain very high accuracies given less than 10% of the parameters of the original ResNet-50.

**Table 9:** Top-1 test accuracy of ResNet-50 on ImageNet.

**Architect**.	**Compression rate**						**Architect**.	**Compression rate**					
	**1%**	**2%**	**5%**	**10%**	**20%**	**50%**		**0.5%**	**1%**	**5%**	**10%**	**20%**	**50%**
NN-CP	57.86%	64.17%	69.37%	71.52%	72.08%	72.44%	TNN-mCP	72.65%	73.76%	74.03%	75.00%	75.31%	77.31%
NN-TT	56.82%	62.23%	65.54%	66.21%	66.90%	66.92%	TNN-mTT	69.27%	73.04%	73.51%	73.50%	73.87%	74.14%
NN-TR	56.59%	62.97%	69.59%	71.61%	73.04%	73.21%	TNN-mTR	67.49%	73.23%	74.12%	75.01%	75.32%	75.16%

*We compare **baseline NNs** against **our TNNs** by **training all models from scratch** (i.e., without reference models). The original ResNet-50 model achieves 78.03% Top-1 test accuracy with 25.6M parameters (He et al., 2016a)*.

**VGG, ResNet and Wide-ResNet with full parameters**. While we use TNNs mostly for model compression in this article, one remaining question is the performance of TNNs when they have the same number of parameters as the original model. To answer this question, we train TNN-mTT *from scratch* with architectures VGG-16 (Simonyan and Zisserman, 2014), ResNet-34 (He et al., 2016b) and WRN-28-10 (Zagoruyko and Komodakis, 2016) on CIFAR-10. As shown in Table 10, TNNs (without hyper-parameter optimization) match/outperform their original model (where the hyper-parameters are highly optimized) when their numbers of parameters are the same.

**Table 10:** Performance of TNNs vs. NNs counterparts on CIFAR-10.

**Acc**.	**TNN VGG**	**NN VGG**	**TNN WRN**	**NN WRN**	**TNN ResNet**	**NN ResNet**
Train	100%	100%	100%	100%	100%	100%
Test	93.68%	92.64%	95.09%	95.83%	91.79%	92.49%

*NN stands for the uncompressed model proposed by the original paper. All models are trained from scratch (i.e., without reference models)*.

## 8. Conclusion

In this work, we introduced a new suite of generalized tensor algebra, which provides systematic notations for generalized tensor operations (a.k.a., tensor networks). Based on these generalized tensor operations, we developed a family of tensorial layers, extending existing fully-connected/convolutional layers in traditional neural networks. We constructed tensorial neural networks (TNNs) using tensorial layers as building blocks, and empirically showed that our TNNs maintain high predictive performance even when they contain significantly fewer parameters than traditional neural networks. Our experiments on LeNet-5, VGG, ResNet, and Wide-ResNet consistently verified that our TNNs outperform the state-of-the-art low-rank architectures under the same compression rate.

## Data Availability Statement

Publicly available datasets were analyzed in this study. This data can be found at: https://www.cs.toronto.edu/~kriz/cifar.html; https://image-net.org/challenges/LSVRC/2012/.

## Author Contributions

JS developed the core ideas for this article under the guidance of FH and implemented all tensorial layers. JL and XL coded the experiments for CIFAR-10 and ImageNet-2012, respectively. TR, CC, and T-CT helped with the experimental design and assisted with the paper writing. All authors contributed to the article and approved the submitted version.

## Funding

This research was supported by a startup fund from the Department of Computer Science at the University of Maryland, the National Science Foundation IIS-1850220 CRII Award 030742-00001, and DOD-DARPA-Defense Advanced Research Projects Agency Guaranteeing AI Robustness against Deception (GARD). FH was also supported by Adobe, Capital One and JP Morgan faculty fellowships.

## Conflict of Interest

The authors declare that the research was conducted in the absence of any commercial or financial relationships that could be construed as a potential conflict of interest.

## Publisher's Note

All claims expressed in this article are solely those of the authors and do not necessarily represent those of their affiliated organizations, or those of the publisher, the editors and the reviewers. Any product that may be evaluated in this article, or claim that may be made by its manufacturer, is not guaranteed or endorsed by the publisher.

## Footnotes

^1^Note that TNN-mTK remains an exception for aggressive compression due to the extreme bottleneck structure that we previously discussed.
