# Supplementary material for: Compact Neural Architecture Designs by Tensor Representations
Source: Front Artif Intell. 2022 Mar 8;5:728761. doi: 10.3389/frai.2022.728761 (PMC8959219; doi:10.3389/frai.2022.728761)
Supplement: Supplementary file 1 [file Data_Sheet_1.pdf]

# Appendix: Compact Neural Architecture Designs by Tensor Representations

**Jiahao Su** [1], **Jingling Li** [2], **Xiaoyu Liu** [2], **Teresa Ranadive** [3], **Christopher Coley** [4], **Tai-Ching Tuan** [3], **Furong Huang** [2,*]

[1] *Department of Electrical and Computer Engineering, University of Maryland, College Park, MD, United States*
[2] *Department of Computer Science, University of Maryland, College Park, MD, United States*
[3] *Laboratory for Physical Sciences, University of Maryland, College Park, MD, United States*
[4] *Department of Aeronautics, United States Air Force Academy, Colorado Springs, CO, United States*

Correspondence*:
Furong Huang
furongh@umd.edu

## A TENSOR DECOMPOSITIONS

Tensor decompositions are generalizations of matrix factorizations to higher-order arrays. We review four commonly used tensor decompositions, namely *canonical polyadic decomposition* [1], *Tucker decomposition* [1], *tensor-train decomposition* [2], and *tensor-ring decomposition* [3], with the tensor algebra introduced in the main text.

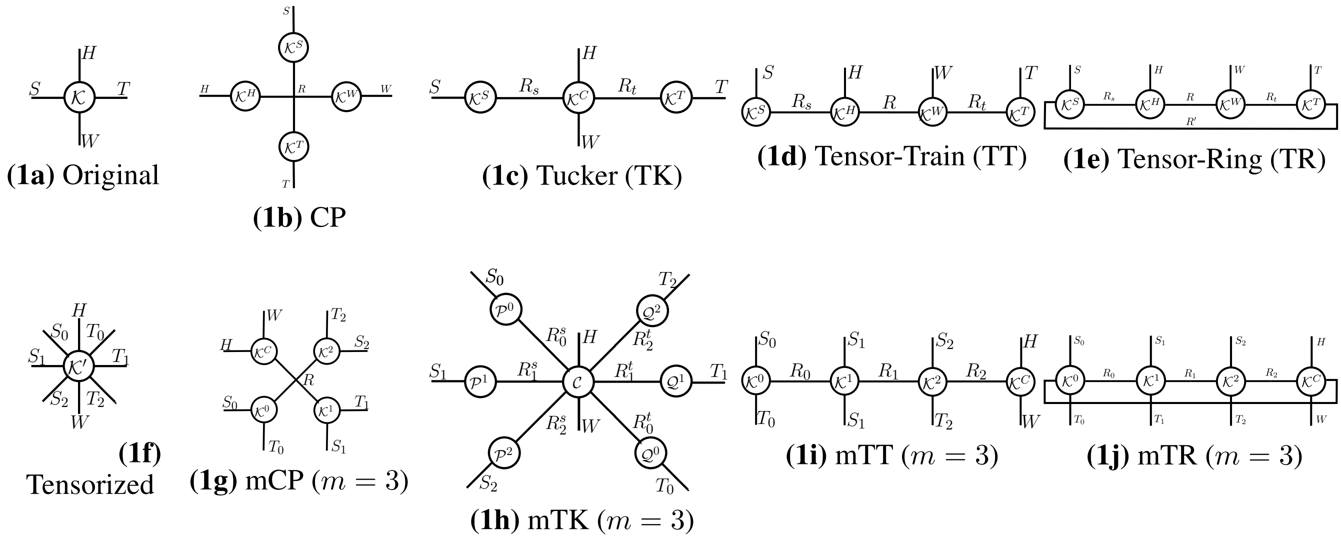


**Figure 1. Diagrams of general tensor decompositions**. Figures **(a)** and **(f)** are the kernels in CNN and TNN respectively. In the first row, Figures **(b)** - **(e)** are four types of tensor decompositions on the convolutional kernel $\mathcal{K}$ in **(a)**. In the second row, Figures **(g)** - **(j)** are four types of general tensor decompositions of the tensorial kernel $\mathcal{K}'$ in **(f)**.

| Type | Definition | Notation | $O$(params.) | $O$(ops.) |
|---|---|---|---|---|
| None | $\mathcal{T}_{i_0,\cdots,i_{m-1}}$ | $\mathcal{T}$ | $I$ | 0 |
| CP | $\sum_{r=0}^{R-1} \mathbf{M}^{(0)}_{r,i_0}\mathbf{M}^{(1)}_{r,i_1}\cdots\mathbf{M}^{(m-1)}_{r,i_{m-1}}$ | $\mathbf{1}\times^R_R\left(\mathbf{M}^{(0)}\otimes^R_R\mathbf{M}^{(1)}\otimes^R_R\cdots\otimes^R_R\mathbf{M}^{(m-1)}\right)$ | $mI^{\frac{1}{m}}R$ | $mIR$ |
| TK | $\sum_{\{r_\ell\}}\mathcal{C}_{r_0,\ldots,r_{m-1}}\mathbf{M}^{(0)}_{r_0,i_0}\cdots\mathbf{M}^{(m-1)}_{r_{m-1},i_{m-1}}$ | $\mathcal{C}\times^{R_0}_{R_0}\mathbf{M}^{(0)}\times^{R_1}_{R_1}\mathbf{M}^{(1)}\cdots\times^{R_{m-2}}_{R_{m-2}}\mathbf{M}^{(m-1)}$ | $R^m+mI^{\frac{1}{m}}R$ | $m^2IR$ |
| TT | $\sum_{\{r_\ell\}}\mathcal{T}^{(0)}_{i_0,r_0}\mathcal{T}^{(1)}_{i_1,r_0,r_1}\cdots\mathcal{T}^{(m-1)}_{i_{m-1},r_{m-2}}$ | $\mathcal{T}^{(0)}\times^{R_0}_{R_0}\mathcal{T}^{(1)}\times^{R_1}_{R_1}\cdots\times^{R_{m-2}}_{R_{m-2}}\mathcal{T}^{(m-1)}$ | $mI^{\frac{1}{m}}R^2$ | $mIR^2$ |
| TR | $\sum_{\{r_\ell\}}\mathcal{T}^{(0)}_{i_0,r_{m-1},r_0}\cdots\mathcal{T}^{(m-1)}_{i_{m-1},r_{m-2},r_{m-1}}$ | $\mathcal{T}^{(0)}\left(\times^{R_0}_{R_0}\circ\times^{R_{m-1}}_{R_{m-1}}\right)\left(\mathcal{T}^{(1)}\times^{R_1}_{R_1}\cdots\mathcal{T}^{(m-1)}\right)$ | $mI^{\frac{1}{m}}R^2$ | $mIR^2$ |

**Table 1. Summary of tensor decompositions.** The table summarizes the definitions and tensor notations for four types of tensor decompositions and lists their numbers of parameters and time complexities to reconstruct the original tensor $\mathcal{T}$. For simplicity, we assume all dimensions $\{I_\ell\}$ of $\mathcal{T}$ are equal, and denote the size of $\mathcal{T}$ as the product of all dimensions $I = \prod_{\ell=0}^{m-1} I_\ell$. Furthermore, we assume all ranks $\{R_\ell\}$ (in TK, TT and TR decompositions) are equal to $R$.

**Canonical polyadic decomposition (CP decomposition)** is a generalization of the singular value decomposition (SVD), which decomposes a tensor into an addition of rank-1 tensors, i.e., outer product of multiple vectors. Specifically, given an $m$-order tensor $\mathcal{T}\in\mathbb{R}^{I_0\times I_1\times\cdots\times I_{m-1}}$, the CP decomposition factorizes it into $m$ factor matrices $\{\mathbf{M}^{(\ell)}\}_{\ell=0}^{m-1}$ with $\mathbf{M}^{(\ell)}\in\mathbb{R}^{R\times I_\ell}, \forall\ell\in[m]$. The *canonical rank* $R$ of the CP decomposition can be larger than $\max_\ell I_\ell$.

$$\mathcal{T}_{i_0,\cdots,i_{m-1}} \triangleq \sum_{r=0}^{R-1}\mathbf{M}^{(0)}_{r,i_0}\mathbf{M}^{(1)}_{r,i_1}\cdots\mathbf{M}^{(m-1)}_{r,i_{m-1}} = \sum_{r=0}^{R-1}\mathbf{M}^{(0)}_{r,:}\otimes\mathbf{M}^{(1)}_{r,:}\otimes\cdots\otimes\mathbf{M}^{(m-1)}_{r,:}, \tag{1a}$$

$$\mathcal{T}\triangleq\mathbf{1}\times^R_R\left(\mathbf{M}^{(0)}\otimes^R_R\mathbf{M}^{(1)}\otimes^R_R\cdots\otimes^R_R\mathbf{M}^{(m-1)}\right), \tag{1b}$$

where $\mathbf{1}\in\mathbb{R}^R$ is a length-$R$ with all ones entries. With the CP decomposition, $\mathcal{T}$ can be represented with only $(R\sum_{\ell=0}^{m-1} I_\ell)$ entries instead of $(\prod_{\ell=0}^{m-1} I_\ell)$ as in the original tensor.

**Tucker decomposition (TK decomposition)** further generalizes the CP decomposition. Given an $m$-order tensor $\mathcal{T}\in\mathbb{R}^{I_0\times I_1\times\cdots\times I_{m-1}}$, the Tucker decomposition factors it into $m$ factor matrices $\{\mathbf{M}^{(\ell)}\}_{\ell=0}^{m-1}$, where $\mathbf{M}^{(\ell)}\in\mathbb{R}^{R_\ell\times I_\ell}, \forall\ell\in[m]$ and an additional $m$-order core tensor $\mathcal{C}\in\mathbb{R}^{R_0\times R_1\times\cdots\times R_{m-1}}$. The *Tucker ranks* $\{R_\ell\}_{\ell=1}^m$ are no greater than the dimensions at their corresponding modes, i.e., $R_\ell\le I_\ell, \forall\ell\in[m]$.

$$\mathcal{T}_{i_0,\cdots,i_{m-1}}\triangleq\sum_{\{r_l\}}\mathcal{C}_{r_0,\ldots,r_{m-1}}\mathbf{M}^{(0)}_{r_0,i_0}\cdots\mathbf{M}^{(m-1)}_{r_{m-1},i_{m-1}}, \tag{2a}$$

$$\mathcal{T}\triangleq\mathcal{C}\times^{R_0}_{R_0}\mathbf{M}^{(0)}\times^{R_1}_{R_1}\mathbf{M}^{(1)}\cdots\times^{R_{m-1}}_{R_{m-1}}\mathbf{M}^{(m-1)}. \tag{2b}$$

Notice that when $R_\ell = R$ for all $\ell\in[m]$ and $\mathcal{C}$ is a super-diagonal tensor with super-diagonal entries being ones, the Tucker decomposition reduces to a CP decomposition in Equation (1). With the Tucker decomposition, a tensor is approximated using $(\prod_{\ell=0}^{m-1} R_\ell + \sum_{\ell=0}^{m-1} I_\ell R_\ell)$ entries.

**Tensor-train decomposition (TT decomposition)** factorizes an $m$-order tensor $\mathcal{T}\in\mathbb{R}^{I_0\times I_1\times\cdots\times I_{m-1}}$ into $m$ low-order tensors $\{\mathcal{T}^{(\ell)}\}_{\ell=0}^{m-1}$ with $\mathcal{T}^{(0)}\in\mathbb{R}^{I_0\times R_0}$, $\mathcal{T}^{(\ell)}\in\mathbb{R}^{I_\ell\times R_{\ell-1}\times R_\ell}$ for $\ell = 1,\cdots,m-2$, and $\mathcal{T}^{(m-1)}\in\mathbb{R}^{I_{m-1}\times R_{m-2}}$.

$$\mathcal{T}_{i_0,\ldots,i_{m-1}}\triangleq\sum_{\{r_\ell\}}\mathcal{T}^{(0)}_{i_0,r_0}\mathcal{T}^{(1)}_{i_1,r_0,r_1}\cdots\mathcal{T}^{(m-1)}_{i_{m-1},r_{m-2}}, \tag{3a}$$

$$\mathcal{T} \triangleq \mathcal{T}^{(0)} \times_{R_0}^{R_0} \mathcal{T}^{(1)} \times_{R_1}^{R_1} \cdots \times_{R_{m-2}}^{R_{m-2}} \mathcal{T}^{(m-1)}. \tag{3b}$$

In the TT decomposition, $\{R_\ell\}_{\ell=1}^m$ are named as the *tensor-train ranks*, which controls the tradeoff between complexity and approximation — the tensor in TT format is represented by $(I_0 R_0 + \sum_{\ell=1}^{m-2} I_\ell R_\ell R_{\ell+1} + I_{m-1} R_{m-1})$ entries.

**Tensor-ring decomposition (TR decomposition)** generalizes the TT decomposition and factorizes an $m$-order tensor $\mathcal{T} \in \mathbb{R}^{I_0 \times I_1 \times \cdots \times I_{m-1}}$ into $m$ low-order tensors $\{\mathcal{T}^{(\ell)}\}_{\ell=0}^{m-1}$ with $\mathcal{T}^{(0)} \in \mathbb{R}^{I_0 \times R_{m-1} \times R_0}$ and $\mathcal{T}^{(\ell)} \in \mathbb{R}^{I_\ell \times R_{\ell-1} \times \times R_\ell}$ for $\ell \geq 1$.

$$\mathcal{T}_{i_0, \dots, i_{m-1}} \triangleq \sum_{\{r_\ell\}} \mathcal{T}^{(0)}_{i_0, r_{m-1}, r_0} \cdots \mathcal{T}^{(m-1)}_{i_{m-1}, r_{m-2}, r_{m-1}}, \tag{4a}$$

$$\mathcal{T} \triangleq \mathcal{T}^{(0)} \left( \times_{R_m}^{R_m} \circ \times_{R_0}^{R_0} \right) \left( \mathcal{T}^{(1)} \times_{R_1}^{R_1} \cdots \mathcal{T}^{(m-1)} \right). \tag{4b}$$

Similarly, the *tensor-ring ranks* $\{R_\ell\}_{\ell=1}^m$ controls the tradeoff between complexity and approximation — the tensor in TR format is represented by $(I_0 R_0 R_{m-1} + \sum_{\ell=1}^{m-1} I_\ell R_{\ell-1} R_\ell$ entries. Notice that when $R_{m-1} = 1$, the TR decomposition reduces to a TT decomposition in Equation (3).

## B TENSORIAL CONVOLUTIONAL LAYERS

In principle, we can use any generalized tensor operation (tensor network) at each layer of a TNN. To limit the search space, we follow the philosophy in Section 6 of the main text to derive the compact architectures. The procedure consists of two steps: **(1)** reshape a standard layer into its higher-order (tensorized) counterpart; **(2)** apply a tensor decomposition in Appendix A.

**Tensorized 2D-convolutional layer** maps a $(m+2)$-order input tensor $\mathcal{U}' \in \mathbb{R}^{X \times Y \times S_0 \times \cdots \times S_{m-1}}$ to another $(m+2)$-order output tensor $\mathcal{V}' \in \mathbb{R}^{X' \times Y' \times T_0 \times \cdots \times T_{m-1}}$ with a $(2m+2)$-order kernel $\mathcal{K}' \in \mathbb{R}^{H \times W \times S_0 \times \cdots \times S_{m-1} \times T_0 \times \cdots \times T_{m-1}}$.

$$\mathcal{V}' = \mathcal{U}' \big( *_X^H \circ *_Y^W \circ \times_{S_0}^{S_0} \circ \cdots \circ \times_{S_{m+1}}^{S_{m+1}} \big) \mathcal{K}'. \tag{5}$$

Notice that the tensorized layer is *equivalent* to a standard 2D-convolutional layer in **??** if $\mathcal{U}'$, $\mathcal{K}'$ and $\mathcal{V}'$ are reshaped from $\mathcal{U}$, $\mathcal{K}$ and $\mathcal{V}$ with $S = \prod_{\ell=0}^{m-1} S_\ell$ and $T = \prod_{\ell=0}^{m-1} T_\ell$.

**mCP-convolutional layer** is derived if we factorize $\mathcal{K}'$ by a modified CP decomposition (Figure 1g).

$$\mathcal{K}' \triangleq \mathbf{1} \times_R^R \left( \mathcal{K}^{(0)} \otimes_R^R \cdots \otimes_R^R \mathcal{K}^{(m)} \right), \tag{6}$$

where $\mathcal{K}^{(\ell)} \in \mathbb{R}^{R \times S_\ell \times T_\ell}, \forall \ell \in [m]$ and $\mathcal{K}^{(m)} \in \mathbb{R}^{R \times H \times W}$ are $(m+1)$ factors. Accordingly, the procedure to evaluate the output $\mathcal{V}'$ consists of $(m+1)$-steps:

$$\mathcal{U}^{(1)} = \mathcal{U}' \times_{S_0}^{S_0} \mathcal{K}^{(0)}, \tag{7a}$$

$$\mathcal{U}^{(\ell+1)} = \mathcal{U}^{(\ell)} \big( \otimes_R^R \circ \times_{S_\ell}^{S_\ell} \big) \mathcal{K}^{(,)} \ell \tag{7b}$$

$$\mathcal{V}' = \mathcal{U}^{(m)} \big( *_H^X \circ *_W^Y \circ \times_R^R \big) \mathcal{K}^{(,)} m \tag{7c}$$

where $\{\mathcal{U}^{(\ell)}\}_{\ell=1}^m$ are $m$ intermediate results. The backpropagation can also be done in $(m+1)$ steps:

$$\frac{\partial \mathcal{L}}{\partial \mathcal{U}^{(m)}} = \frac{\partial \mathcal{L}}{\partial \mathcal{V}} \big( *_H^{X'^\top} \circ *_W^{Y'^\top} \big) \mathcal{K}^{(m)}, \tag{8a}$$

$$\frac{\partial \mathcal{L}}{\partial \mathcal{K}^{(m)}} = \frac{\partial \mathcal{L}}{\partial \mathcal{V}'} \left( *_X^{X'^\top} \circ *_Y^{Y'^\top} \times_{T_0}^{T_0} \circ \cdots \circ \times_{T_{m-1}}^{T_{m-1}} \right) \mathcal{U}^{(m)}, \tag{8b}$$

$$\frac{\partial \mathcal{L}}{\partial \mathcal{U}^{(\ell)}} = \frac{\partial \mathcal{L}}{\partial \mathcal{U}^{(\ell+1)}} \left( \otimes_R^R \circ \times_{T_\ell}^{T_\ell} \right) \mathcal{K}^{(\ell)}, \tag{8c}$$

$$\frac{\partial \mathcal{L}}{\partial \mathcal{K}^{(\ell)}} = \frac{\partial \mathcal{L}}{\partial \mathcal{U}^{(\ell+1)}} \left( \otimes_R^R \circ \times_X^X \circ \times_Y^Y \circ \times_{S_0}^{S_0} \circ \cdots \circ \times_{T_{\ell-1}}^{T_{\ell-1}} \circ \times_{S_{\ell+1}}^{S_{\ell+1}} \circ \cdots \circ \times_{S_{m-1}}^{S_{m-1}} \right) \mathcal{U}^{(\ell)}, \tag{8d}$$

$$\frac{\partial \mathcal{L}}{\partial \mathcal{U}'} = \frac{\partial \mathcal{L}}{\partial \mathcal{U}^{(1)}} \left( \times_R^R \circ \times_{T_0}^{T_0} \right) \mathcal{K}^{(0)}, \tag{8e}$$

$$\frac{\partial \mathcal{L}}{\partial \mathcal{K}^{(0)}} = \frac{\partial \mathcal{L}}{\partial \mathcal{U}^{(1)}} \left( \times_X^X \circ \times_Y^Y \circ \times_{S_1}^{S_1} \circ \cdots \circ \times_{S_{m-1}}^{S_{m-1}} \right) \mathcal{U}'. \tag{8f}$$

**mTK-convolutional layer** is derived if we factorize $\mathcal{K}'$ by a modified TK decomposition (Figure 1h).

$$\mathcal{K}' \triangleq \mathcal{C} \times_{R_0^s}^{R_0^s} \mathcal{P}^{(0)} \cdots \times_{R_{m-1}^s}^{R_{m-1}^s} \mathcal{P}^{(m-1)} \times_{R_0^t}^{R_0^t} \mathcal{Q}^{(0)} \cdots \times_{R_{m-1}^t}^{R_{m-1}^t} \mathcal{Q}^{(m-1)}, \tag{9}$$

where $\mathcal{P}^{(\ell)} \in \mathbb{R}^{S_\ell \times R_\ell^s}$, $\mathcal{Q}^{(\ell)} \in \mathbb{R}^{R_\ell^t \times T_\ell}$ for $\ell = 1, \cdots, m$ and $\mathcal{C} \in \mathbb{R}^{H \times W \times R_0^s \times \cdots \times R_{m-1}^s \times R_0^t \times \cdots \times R_{m-1}^t}$ are named as input factors, output factors, and core factor respectively. The forward pass of an mTK-convolutional layer takes three steps:

$$\mathcal{U}^{(0)} = \mathcal{U}' \times_{S_0}^{S_0} \mathcal{P}^{(0)} \cdots \times_{S_{m-1}}^{S_{m-1}} \mathcal{P}^{(m-1)}, \tag{10a}$$

$$\mathcal{U}^{(1)} = \mathcal{U}^{(0)} \left( *_H^X \circ *_W^Y \circ \times_{R_0^s}^{R_0^s} \circ \cdots \circ \times_{R_{m-1}^s}^{R_{m-1}^s} \right) \mathcal{C}, \tag{10b}$$

$$\mathcal{V}' = \mathcal{U}^{(1)} \times_{R_0^t}^{R_0^t} \mathcal{Q}^{(0)} \cdots \times_{R_{m-1}^t}^{R_{m-1}^t} \mathcal{Q}^{(m-1)}, \tag{10c}$$

where $\mathcal{U}^{(0)}, \mathcal{U}^{(1)}$ are intermediate results. The backpropagation through the intermediate tensors are

$$\frac{\partial \mathcal{L}}{\partial \mathcal{U}^{(1)}} = \frac{\partial \mathcal{L}}{\partial \mathcal{V}'} \times_{T_0}^{T_0} \mathcal{Q}^{(0)} \cdots \times_{T_{m-1}}^{T_{m-1}} \mathcal{Q}^{(m-1)}, \tag{11a}$$

$$\frac{\partial \mathcal{L}}{\partial \mathcal{U}^{(0)}} = \frac{\partial \mathcal{L}}{\partial \mathcal{U}^{(1)}} \left( *_H^{X'^\top} \circ *_W^{Y'^\top} \circ \times_{R_0^t}^{R_0^t} \circ \cdots \circ \times_{R_{m-1}^t}^{R_{m-1}^t} \right) \mathcal{C}, \tag{11b}$$

$$\frac{\partial \mathcal{L}}{\partial \mathcal{U}'} = \frac{\partial \mathcal{L}}{\partial \mathcal{U}^{(0)}} \times_{R_0^s}^{R_0^s} \mathcal{P}^{(0)} \cdots \times_{R_{m-1}^s}^{R_{m-1}^s} \mathcal{P}^{(m-1)}, \tag{11c}$$

with the gradient with respect to $\mathcal{C}$ as

$$\frac{\partial \mathcal{L}}{\partial \mathcal{C}} = \frac{\partial \mathcal{L}}{\partial \mathcal{U}^{(1)}} \left( *_X^{X'^\top} \circ *_{Y'}^{Y'^\top} \right) \mathcal{U}^{(0)}. \tag{12}$$

**mTT-convolutional layer** is derived if we factorize $\mathcal{K}'$ by a modified TT decomposition (Figure 1i).

$$\mathcal{K}' \triangleq \mathcal{K}^{(0)} \times_{R_0}^{R_0} \mathcal{K}^{(1)} \times_{R_1}^{R_1} \cdots \times_{R_{m-1}}^{R_{m-1}} \mathcal{K}^{(m)}, \tag{13}$$

where $\mathcal{K}^{(0)} \in \mathbb{R}^{S_0 \times T_0 \times R_0}$, $\mathcal{K}^{(l)} \in \mathbb{R}^{R_{\ell-1} \times S_\ell \times T_\ell \times R_\ell}$ for $\ell = 1, \cdots, m$, and $\mathcal{K}^{(m)} \in \mathbb{R}^{R_{m-1} \times H \times W}$ are $(m+1)$ factors. The forward pass to evaluate $\mathcal{V}$ consists of $(m+1)$ steps:

$$\mathcal{U}^{(1)} = \mathcal{U}' \times_{S_0}^{S_0} \mathcal{K}^{(0)}, \tag{14a}$$

$$\mathcal{U}^{(\ell+1)} = \mathcal{U}^{(\ell)}\left( \times^{R_{\ell-1}}_{R_{\ell-1}} \circ \times^{S_\ell}_{S_\ell} \right)\mathcal{K}^{(\ell)}, \tag{14b}$$

$$\mathcal{V}' = \mathcal{U}^{(m)}\left( *^{X}_{H} \circ *^{Y}_{W} \circ \times^{R_{m-1}}_{R_{m-1}} \right)\mathcal{K}^{(m)}, \tag{14c}$$

where $\mathcal{U}^{(\ell)} \in \mathbb{R}^{X \times Y \times S_\ell \times \cdots \times S_{m-1} \times T_0 \times \cdots \times T_{\ell-1} \times R_{\ell-1}}$ with $\ell = 1, \cdots, m$ are intermediate results. We summarize their backpropagation equations as follows:

$$\frac{\partial \mathcal{L}}{\partial \mathcal{U}^{(m)}} = \frac{\partial \mathcal{L}}{\partial \mathcal{V}}\left( *^{X'^\top}_{H} \circ *^{Y'^\top}_{W} \right)\mathcal{K}^{(m)} \tag{15a}$$

$$\frac{\partial \mathcal{L}}{\partial \mathcal{K}^{(m)}} = \frac{\partial \mathcal{L}}{\partial \mathcal{V}'}\left( *^{X'^\top}_{X} \circ *^{Y'^\top}_{Y} \times^{T_0}_{T_0} \circ \cdots \circ \times^{T_{m-1}}_{T_{m-1}} \right)\mathcal{U}^{(m)}, \tag{15b}$$

$$\frac{\partial \mathcal{L}}{\partial \mathcal{U}^{(\ell)}} = \frac{\partial \mathcal{L}}{\partial \mathcal{U}^{(\ell+1)}}\left( \times^{R_\ell}_{R_\ell} \circ \times^{T_\ell}_{T_\ell} \right)\mathcal{K}^{(\ell)}, \tag{15c}$$

$$\frac{\partial \mathcal{L}}{\partial \mathcal{K}^{(\ell)}} = \frac{\partial \mathcal{L}}{\partial \mathcal{U}^{(\ell+1)}}\left( \times^{X}_{X} \circ \times^{Y}_{Y} \circ \times^{S_0}_{S_0} \circ \cdots \circ \times^{T_{\ell-1}}_{T_{\ell-1}} \circ \times^{S_{\ell+1}}_{S_{\ell+1}} \circ \cdots \circ \times^{S_{m-1}}_{S_{m-1}} \right)\mathcal{U}^{(\ell)}, \tag{15d}$$

$$\frac{\partial \mathcal{L}}{\partial \mathcal{U}'} = \frac{\partial \mathcal{L}}{\partial \mathcal{U}^{(1)}}\left( \times^{R_0}_{R_0} \circ \times^{T_0}_{T_0} \right)\mathcal{K}^{(0)}, \tag{15e}$$

$$\frac{\partial \mathcal{L}}{\partial \mathcal{K}^{(0)}} = \frac{\partial \mathcal{L}}{\partial \mathcal{U}^{(1)}}\left( \times^{X}_{X} \circ \times^{Y}_{Y} \circ \times^{S_1}_{S_1} \circ \cdots \circ \times^{S_{m-1}}_{S_{m-1}} \right)\mathcal{U}'. \tag{15f}$$

**mTR-convolutional layer** is derived if we factorize $\mathcal{K}'$ by a modified TR decomposition (Figure 1i).

$$\mathcal{K}' \triangleq \mathcal{K}^{(0)}\left( \times^{R_m}_{R_m} \circ \times^{R_0}_{R_0} \right)\left( \mathcal{K}^{(1)} \times^{R_1}_{R_1} \cdots \times^{R_{m-1}}_{R_{m-1}} \mathcal{K}^{(m)} \right), \tag{16}$$

where $\mathcal{K}^{(0)} \in \mathbb{R}^{S_0 \times T_0 \times R_m \times R_0}$, $\mathcal{K}^{(\ell)} \in \mathbb{R}^{R_{\ell-1} \times S_\ell \times T_\ell \times R_\ell}$ for $\ell = 1, \cdots, m$, and $\mathcal{K}^{(m)} \in \mathbb{R}^{R_{m-1} \times R_m \times H \times W}$ are $(m+1)$ factors. The forward pass to evaluate $\mathcal{V}$ also has $(m+1)$ steps:

$$\mathcal{U}^{(1)} = \mathcal{U}' \times^{S_0}_{S_0} \mathcal{K}^{(0)}, \tag{17a}$$

$$\mathcal{U}^{(\ell+1)} = \mathcal{U}^{(\ell)}\left( \times^{R_{\ell-1}}_{R_{\ell-1}} \circ \times^{S_\ell}_{S_\ell} \right)\mathcal{K}^{(l)}, \tag{17b}$$

$$\mathcal{V}' = \mathcal{U}^{(m)}\left( *^{X}_{H} \circ *^{Y}_{W} \circ \times^{R_{m-1}}_{R_{m-1}} \circ \times^{R_m}_{R_m} \right)\mathcal{K}^{(m)}, \tag{17c}$$

where $\mathcal{U}^{(l)} \in \mathbb{R}^{X \times Y \times S_\ell \times \cdots \times S_{m-1} \times T_0 \times \cdots \times T_{\ell-1} \times R_{m-1} \times R_{\ell-1}}, \forall \ell = 1, \cdots, m$ are $m$ intermediate results. We summarize their backpropagation equations as follows:

$$\frac{\partial \mathcal{L}}{\partial \mathcal{U}^{(m)}} = \frac{\partial \mathcal{L}}{\partial \mathcal{V}}\left( *^{X'^\top}_{H} \circ *^{Y'^\top}_{W} \right)\mathcal{K}^{(m)}, \tag{18a}$$

$$\frac{\partial \mathcal{L}}{\partial \mathcal{K}^{(m)}} = \frac{\partial \mathcal{L}}{\partial \mathcal{V}'}\left( *^{X'^\top}_{X} \circ *^{Y'^\top}_{Y} \times^{T_0}_{T_0} \circ \cdots \circ \times^{T_{m-1}}_{T_{m-1}} \right), \mathcal{U}^{(m)} \tag{18b}$$

$$\frac{\partial \mathcal{L}}{\partial \mathcal{U}^{(\ell)}} = \frac{\partial \mathcal{L}}{\partial \mathcal{U}^{(\ell+1)}}\left( \times^{R_\ell}_{R_\ell} \circ \times^{T_\ell}_{T_\ell} \right)\mathcal{K}^{(\ell)}, \tag{18c}$$

$$\frac{\partial \mathcal{L}}{\partial \mathcal{K}^{(\ell)}} = \frac{\partial \mathcal{L}}{\partial \mathcal{U}^{(\ell+1)}}\left( \times^{X}_{X} \circ \times^{Y}_{Y} \circ \times^{S_0}_{S_0} \circ \cdots \circ \times^{T_{\ell-1}}_{T_{\ell-1}} \circ \times^{S_{\ell+1}}_{S_{\ell+1}} \circ \cdots \circ \times^{S_{m-1}}_{S_{m-1}} \right)\mathcal{U}^{(l)}, \tag{18d}$$

$$\frac{\partial \mathcal{L}}{\partial \mathcal{U}'} = \frac{\partial \mathcal{L}}{\partial \mathcal{U}^{(1)}}\left( \times^{R_m}_{R_m} \circ \times^{R_0}_{R_0} \circ \times^{T_0}_{T_0} \right)\mathcal{K}^{(0)}, \tag{18e}$$

$$\frac{\partial \mathcal{L}}{\partial \mathcal{K}^{(0)}} = \frac{\partial \mathcal{L}}{\partial \mathcal{U}^{(1)}} \left( \times_X^X \circ \times_Y^Y \circ \times_{S_1}^{S_1} \circ \cdots \circ \times_{S_{m-1}}^{S_{m-1}} \right) \mathcal{U}'. \tag{18f}$$

## REFERENCES

[1] Kolda TG, Bader BW. Tensor decompositions and applications. *SIAM review* **51** (2009) 455–500.

[2] Oseledets IV. Tensor-train decomposition. *SIAM Journal on Scientific Computing* **33** (2011) 2295–2317.

[3] Zhao Q, Zhou G, Xie S, Zhang L, Cichocki A. Tensor ring decomposition. *arXiv preprint arXiv:1606.05535* (2016).
